# Probiotics Supplementation during Pregnancy: Can They Exert Potential Beneficial Effects against Adverse Pregnancy Outcomes beyond Gestational Diabetes Mellitus?

**DOI:** 10.3390/biology13030158

**Published:** 2024-02-28

**Authors:** Efthymios Poulios, Eleni Pavlidou, Sousana K. Papadopoulou, Kalliopi Rempetsioti, Athanasios Migdanis, Maria Mentzelou, Maria Chatzidimitriou, Ioannis Migdanis, Odysseas Androutsos, Constantinos Giaginis

**Affiliations:** 1 Department of Food Science and Nutrition, School of the Environment, University of the Aegean, 81400 Lemnos, Greece; epoulios@aegean.gr (E.P.); elen.p.pavl@gmail.com (E.P.); fns16105@fns.aegean.gr (K.R.); maria.mentzelou@hotmail.com (M.M.); 2 Department of Nutritional Sciences and Dietetics, Faculty of Health Sciences, International Hellenic University, 57400 Thessaloniki, Greece; souzpapa@gmail.com; 3 Faculty of Medicine, University of Thessaly, 41500 Larissa, Greece; thanmig@yahoo.com (A.M.); johnymig@hotmail.com (I.M.); 4 Department of Nutrition and Dietetics, School of Physical Education, Sport Science and Dietetics, University of Thessaly, 42132 Trikala, Greece; oandroutsos@uth.gr; 5 Department of Biomedical Science, International Hellenic University, 57400 Thessaloniki, Greece; chdimitr@ihu.gr

**Keywords:** probiotics, pregnancy, adverse pregnancy outcomes, functional properties, health claims, supplementation

## Abstract

**Simple Summary:**

In the last few years, several clinical trials have evaluated the potential positive effects of probiotics supplementation against several adverse pregnancy outcomes. However, it is currently questionable whether the existing studies are adequate to derive potential recommendations of high validity and accuracy. From this perspective, the aim of the current narrative review focuses on the critical summarization and scrutinization of the currently available clinical studies by searching the most precise international databases, such as PubMed, Scopus, and Web of Sciences. Most of the existing evidence seems promising, rendering probiotics as potential effective preventive factors and/or co-treatment agents against diverse adverse pregnancy outcomes. However, there are several limitations and high heterogeneity amongst the existing clinical studies to be considered in order to derive specific recommendations of high validity to introduce probiotics supplementation in the clinical practice. Thus, further research is highly recommended in order to obtain more adequate conclusions.

**Abstract:**

Background: Probiotics, as supplements or food ingredients, are considered to exert promising healthy effects when administered in adequate quantity. Probiotics’ healthy effects are related with the prevention of many diseases, as well as decreasing symptom severity. Currently, the most available data concerning their potential health effects are associated with metabolic disorders, including gestational diabetes mellitus. There is also clinical evidence supporting that they may exert beneficial effects against diverse adverse pregnancy outcomes. The purpose of the current narrative study is to extensively review and analyze the current existing clinical studies concerning the probable positive impacts of probiotics supplementation during pregnancy as a protective agent against adverse pregnancy outcomes beyond gestational diabetes mellitus. Methods: a comprehensive and thorough literature search was conducted in the most precise scientific databases, such as PubMed, Scopus, and Web of Sciences, utilizing efficient, representative, and appropriate keywords. Results: in the last few years, recent research has been conducted concerning the potential beneficial effects against several adverse pregnancy outcomes such as lipid metabolism dysregulation, gestational hypertensive disorders, preterm birth, excessive gestational weight gain, caesarean risk section, vaginal microbiota impairment, mental health disturbances, and others. Conclusion: up to the present day, there is only preliminary clinical data and not conclusive results for probiotics’ healthy effects during pregnancy, and it remains questionable whether they could be used as supplementary treatment against adverse pregnancy outcomes beyond gestational diabetes mellitus.

## 1. Introduction

Probiotics, as supplements or food products, have been shown to promote human health when administered in appropriate quantities. The most utilized strains, belonging to the class of *Bifidobacterium* and *Lactobacillus*, can considerably influence various aspects of human health through diverse molecular mechanisms [1,2]. The minimum effective probiotic concentrations remain insufficient [1,2]. Based on the Food and Agriculture Organization of the United Nations (FAO) and the World Health Organization (WHO), it is generally accepted that probiotic products should include a minimum concentration of 10^6^ CFUs/mL or gram and that a total of some 10^8^ to 10^9^ probiotic microorganisms should be consumed per day in order for the probiotic impact to be transferred to the consumer [1,2]. The FAO/WHO definition of probiotics can be interpreted into four simple and rational criteria permitting one to decide if specific strains of microorganisms qualify as a probiotic for usage in foods and nutritional supplements [3]. Probiotic strains should be (a) adequately characterized, (b) safe for the planned usage, (c) supported by at least one positive human clinical trial based on commonly recognized scientific standards or as per recommendations and provisions of local/national authorities when applicable, and (d) active in the product at an effective dose throughout shelf life [3].

Probiotics may act as preventing agents against several bacterial pathogens by limiting binding locations on mucosal epithelial cells and regulating the host immunological response, therefore enhancing the gut barrier [4]. The diverse strengths of probiotics are also associated with the control of the intestinal microbiome, justification of dietary intolerances (like lactose intolerance), bioavailability enhancement of macro- and micro-nutrients, and weakening of allergic symptomatology in vulnerable persons [5]. These beneficial impacts of probiotics are mainly associated with the prevention of several disorders by decreasing symptom severity [1,2,3,4,5]. There is currently substantial evidence that probiotic supplementation could act as a protective agent against allergic symptoms, tumor malignancies, neurodegenerative and psychiatric diseases, cardiovascular diseases, obesity, diabetes, lactose intolerance, inflammatory bowel disease, immune system disorders, non-alcoholic fatty liver disease, diarrhea, irritable bowel syndrome, etc. [6,7,8,9,10,11,12,13,14]. However, there is not at this time adequate conclusive evidence concerning their potential health effects, and it remains questionable whether they may be applied as supplementary treatment in any disease.

Currently, the most available data concerning their potential health effects are related with metabolic disorders, including gestational diabetes mellitus (GDM) [15,16]. Notably, the gut bacteria ecosystem has been well recognized to exert a crucial impact in controlling energy homeostasis and is highly related with the risk of metabolism disorders [15,16]. In the last few years, there have been cumulative clinical studies investigating the probable positive effects of probiotics supplements in preventing and/or co-treating GDM [17,18,19,20]. Even if there is heterogeneity among the existing clinical studies, most of them have supported that probiotics supplementation may decrease the risk of GDM by reducing its influencing metabolism characteristics, including blood glucose concentrations, lipids levels, inflammatory conditions, and oxidation biomarkers, all of which could lower GDM incidence among pregnant women [17,18,19,20]. GDM constitutes one of the most common perinatal risk factors which may negatively affect both the mother’s and fetus’ health, exerting severe short- and long-term harmful pregnancy effects if remained untreated [21,22]. Beyond GDM, pregestational maternal obesity, excessive gestational weight gain (GWG), gestational hypertensive disorders, macrosomia or low birth weight, preterm birth, lipid metabolism dysfunction, and fetal growth restriction within gestation could result in harmful pregnancy effects and complications [21,22,23,24,25,26].

Although there are several review articles focused on GDM, there are not any recent comprehensive literature reviews for the other adverse pregnancy outcomes. In light of the above, the present study aims to comprehensively summarize and analyze the potential beneficial effects of probiotics supplementation against adverse pregnancy outcomes beyond GDM.

## 2. Methods

A thorough search was conducted in the most precise and reliable international databases, such as PubMed, Scopus, and Web of Sciences, from January 2000 to December 2023, utilizing efficient, characteristic, and representative keywords like probiotics, prebiotics, nutritional interventions, gut microbiome, gut flora, microorganisms, obesity, childbirth, excessive GWG, obesity, gestational hypertensive disorders, pregnancy adverse outcomes, preterm birth, lipid metabolism, caesarean section, vaginal microbiota, etc. We included surveys using exclusively English language, clinical human surveys, and randomized clinical trials (RCTs), as well as systematic reviews and meta-analysis studies. Gray literature, commentaries, editorials, editor letters, in vitro and in vivo animal surveys, and abstracts in congresses’ proceedings, as well as papers published in non-peer-reviewed journals were not included in the final analysis.

To enhance the validity of this research, all authors acted as reviewers. All reviewers screened the retrieved published articles, evaluated the reported results, and adjusted the screening and information extraction manually prior to beginning screening. All reviewers worked in groups of two and sequentially evaluated the titles, abstracts, and then the corresponding full texts of all publications identified as additional, relevant publications, ensuring a well-organized methodology and a reliable study design. We solved arguments on survey selection and data extraction through agreement by and discussion with all of the authors/reviewers. A data chart form was established by two reviewers (E.P.,Efthymios Poulios, and C.G.) who separately recorded the data, conferred the findings, and constantly reorganized the data chart form.

**Figure 1:**
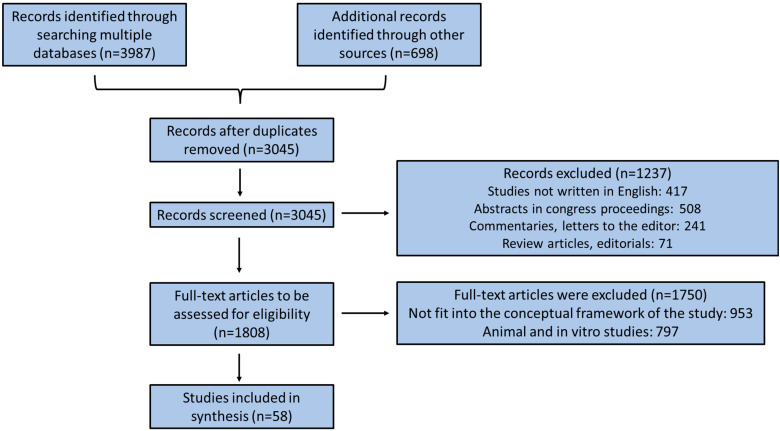
Flow chart diagram to select the final surveys contained in the analysis.

The search was extended by searching the citation records of the appropriate collected surveys, as well as hand-searching relevant journals, commentaries, editorials, and abstracts in congresses’ proceedings for relevant incited studies. The recovered studies were further carefully tested for associated studies cited in their manuscript. Non-English articles, non-human trials, non-full-text studies in languages other than English, and duplicate reports were excluded from the study. The included studies were chosen according to their suitability, and the most appropriate ones were selected and reported below based on the flow chart diagram illustrated in Figure 1.

## 3. Results

By applying the aforementioned inclusion and exclusion criteria, we identified 58 clinical human studies, which were analyzed in the present narrative review. Ten of them simultaneously included two methodological designs, and therefore, they were considered as double clinical studies. Specifically, we retrieved 44 RCTs, 4 prospective cohort studies, 3 retrospective studies, 2 pilot studies, and 15 meta-analysis studies. Most of them (n = 16) were performed in Iran, six studies were conducted in China, while Finland, Canada, and New Zealand carried out four studies each. Italy, Malaysia, Norway, and Australia contributed three studies each. The Netherlands and Japan performed two studies each. Taiwan, Israel, Thailand, Austria, Rwanda, Ireland, Spain, and Germany conducted one study each. Twenty-three clinical studies were included as a study sample of pregnant women with GDM and twelve clinical studies were conducted with overweight and/or obesity pregnant women. Lastly, 18 clinical studies were included as a study sample of pregnant women without any diseases during their pregnancy. The vast majority of the analyzed studies used probiotic supplements Lactobacillus and Bifidobacterium species. Most of the included studies used a probiotic dose ranging from 10^9^ to 10^11^ CFUs/capsule with a mean interventional duration of approximately 10 weeks.

### 3.1. Hypertensive Disorders during Gestation

Hypertensive disorders during gestation show a prevalence of 5–10% concerning all gestations and this prevalence appears to constantly be increasing globally [27,28]. Hypertensive disorders during gestation are mainly classified into four categories: chronic (pre-existing) hypertension, gestational hypertension or pregnancy-induced hypertensive conditions, pre-eclampsia/eclampsia, and pre-eclampsia overlaid on long-lasting hypertension [29,30]. Among them, pre-eclampsia constitutes one of the most common medical diseases affecting gestation related with considerable mother and fetus disease and death rates. The most severe maternal problems of pre-eclampsia include intracerebral hemorrhage, eclampsia, and kidney dysfunction, as well as hemolysis, enhanced hepatic enzymes, decreased prevalence of platelets syndrome, and subsequent revocable encephalopathy syndrome [29,30]. Currently, there are various risk factors which have been related with gestational hypertension, such as women with overweight/obesity before gestation, older women, poor physical activity, systematic smoking, enhanced alcohol drinking, and a history of hypertension [31,32,33]. The probable positive effects of probiotic supplements in preventing or co-treating gestational hypertensive disorders are presented in Table 1.

Among the first population-based studies, a Norwegian study including 33,399 primiparous pregnant women without chronic hypertension was performed. In this study, daily consumption of milk food stuffs including probiotics during the intermediate months of gestation was related with lower probability of developing pre-eclampsia [34]. This relation was most pronounced in advanced pre-eclampsia, while an uncertain dose-dependent protection with elevated consumption compared to no consumption was noted [34]. This observational study had several strengths as it included a large sample of nulliparous women enrolled from both urban and rural areas and including all age and socio-economic classes. The above survey also had a prospective methodology, and data concerning nutrient consumption and probable confounding factors were gathered prior to the onset of pre-eclampsia [34]. However, dietary intake of probiotic milk products was assessed only once during pregnancy, and there may have been changes in consumption that were not registered. It should be noted that the association between probiotic intake and severe pre-eclampsia was stronger in women with a body mass index (BMI) of more than 25 compared to the group with a BMI of 25 or lower [35]. In this context, it was previously shown that the supernatant of the probiotic bacteria *Lactobacillus rhamnosus* GR-1 modified the lipopolysaccharide inflammatory response in placental trophoblast cells, a potential key cell type in pre-eclampsia [36].

**Table 1:** Clinical studies evaluating the impact of probiotic supplements on the prevention or co-treatment of gestational hypertensive disorders.

Type of Study	Study Population	Probiotics Treatment	Main Findings	Ref.
Prospective Norwegian Mother and Child Cohort Study	Pregnant women, n = 33,399	Milk-related products containing probiotic lactobacilli (*Lactobacillus acidophilus*, *Bifidobacterium lactis*, and *Lactobacillus rhamnosus* GG; *Lactobacillus acidophilus* LA-5 and B. lactis) at a concentration range from 1.4 × 10^10^ to 1.6 × 10^11^ bacteria/mL during pregnancy.	A reduced risk of developing pre-eclampsia. The relation of probiotic consumption with severe pre-eclampsia was higher in female participants with BMI ˃ 25 Kg/m^2^ than those with BMI ˂ 25 Kg/m^2^.	[34]
Prospective Norwegian Mother and Child Cohort Study	Pregnant, women, n = 37,050	Probiotic milk intake (*Lactobacillus acidophilus*, *Lactobacillus rhamnosus,* and *Bifidobacterium lactis*) at a concentration range from 1.4 × 10^10^ to 1.6 × 10^11^ bacteria/mL during pregnancy.	A significant association with lower pre-eclampsia risk during the last months of gestation (but not before or during the initial months of gestation) was recorded.	[36]
Double-blind placebo RCT	Pregnant women with GDM, n = 90	Synbiotic capsule, including *Lactobacillus acidophilus*, *Lactobacillus plantarum*, *Lactobacillus fermentum*, and *Lactobacillus gasseri* (1.5–7.0 × 10^9−10^ CFUs/g), with fructooligosaccharide (38.5 mg), or placebo for a duration of 6 weeks.	A significant decrease in systolic and diastolic blood pressure in synbiotic participants compared to those taking the placebo was noted.	[37]
Double-blind, placebo-controlled RCT	Pregnant women with GDM, n = 64	Probiotic capsules containing 180 mg (>4 × 10^9^ CFUs) of *Lactobacillus acidophilus*, *Bifidobacterium, Streptococcus thermophilus,* and *Lactobacillus delbrueckii bulgaricus* plus dextrose anhydride filler and magnesium stearate lubrica for 8 weeks.	Probiotic supplements prevented an increase in systolic blood pressure and decreased diastolic blood pressure.	[38]
Double-blind, placebo-controlled RCT in Iran	Pregnant women with GDM, n = 60	*Lactobacillus acidophilus*, *Lactobacillus casei,* and *Bifidobacterium bifidum* (2 × 10^9^ CFUs/g each) for a duration of 6 weeks.	Probiotic supplementation did not affect any pregnancy outcomes, including gestational hypertensive disorders.	[39]
Double-blind placebo-controlled RCT	149 pregnant women with GDM	Daily probiotic (*Lactobacillus salivarius* at a target dose of 10^9^ CFUs) or placebo from diagnosis until childbirth.	Probiotics were not found to exert any considerably impact on the incidence of pre-eclampsia.	[40]
A meta-analysis of nine RCTs	Pregnant women with GDM, n = 695	Several probiotics were used. The two most received were *Lactobacillus* and *Bifidobacterium* species at a dose of about 10^9^ CFUs for 6 to 8 weeks.	The prevalence of gestational hypertension was not different between the probiotic and placebo groups.	[41]
A meta-analysis of five RCTs	Pregnant women with GDM, n = 402	Probiotic or synbiotic supplementation (*Lactobacillus*, *Bifidobacterium*, and *Streptococcus* species) at a dose from about 1 × 10^9^ to 5 × 10^10^ CFUs for 6 to 8 weeks.	Systolic and diastolic blood pressure and pre-eclampsia were not affected by probiotic administration.	[42]
A meta-analysis of five RCTs	Overweight or obese pregnant women, n = 1048	All studies administrated diverse species of *Lactobacillus* and *Bifidobacterium* at a dose from 1 × 10^9^ to 5 × 10^10^ CFUs for 4 to 6 weeks.	Probiotics may enhance the probability of pre-eclampsia, including superimposed.	[43]

RCTs: randomized clinical trials, GDM: gestational diabetes mellitus, CFUs: colony-forming units, and BMI: body mass index.

Furthermore, Nordqvist et al. conducted an observational study to examine if the scheduling of probiotic milk consumption prior to or during early or late gestation may influence the risk of pre-eclampsia [36]. This study showed that probiotic-containing milk intake during the last months of gestation was related with a reduced probability of pre-eclampsia, especially advanced pre-eclampsia. Nevertheless, no relation was noted between probiotic administration prior to or in the initial months of gestation and pre-eclampsia risk [36]. Moreover, in a double-blind placebo RCT, 90 pregnant women with GDM were enrolled to consume a synbiotic capsule each day including *Lactobacillus acidophilus*, *Lactobacillus plantarum*, *Lactobacillus fermentum*, and *Lactobacillus gasseri* with fructooligosaccharide for 6 weeks [37]. A significant decrease in systolic and diastolic blood pressure in the synbiotic group compared to the placebo group was noted [37]. Accordingly, Hajufaraju et al. performed a double-blind RCT in 64 pregnant women with GDM over 8 weeks [38]. In this study, a probiotic supplement was used which contained 180 mg (>4 × 10^9^ CFUs) of a standard powder, containing freeze-dried *Lactobacillus acidophilus* LA-5, *Bifidobacterium* BB-12, *Streptococcus thermophilus* STY-31, and *Lactobacillus delbrueckii bulgaricus* LBY-27, plus dextrose anhydride filler and magnesium stearate lubrica [38]. This study clearly indicated that intake of probiotics supplementation for 8 weeks blocked an enhancement in systolic blood pressure and reduced diastolic blood pressure in pregnant women with GDM [38].

In view of the above considerations, it should be noted that the surveys which utilized more species like *Lactobacillus* in conjunction with *Bifidobacterium* documented greater beneficial results [37,38]. For instance, the decreases in systolic and diastolic blood pressure in pregnant women with GDM in the survey by Hajifaraji et al. [38] which utilized both probiotic strains were more considerable compared with the survey by Nabhani et al. [37] which utilized only one Lactobacillus species [37,38]. Moreover, in the survey by Hajifaraji et al. [38], who evaluated the results every two weeks until the fourth week, no considerable variation in systolic and diastolic blood pressure between the two groups was noted. From the sixth week, the probiotic impact was considerable, and from eighth week, the statistical significance remained very elevated [38].

Badehnoosh et al. enrolled 60 pregnant women with GDM assigned to receive either one synbiotic capsule including *Lactobacillus acidophilus*, *Lactobacillus casei*, and *Bifidobacterium bifidum* (2 × 10^9^ CFUs/g each) or a placebo for a period of six weeks. This study showed that probiotics supplementation led to a considerable reduction in fasting plasma glucose, hs-CRP, plasma MDA concentrations, and MDA/TAC ratio, as well as substantially enhanced TAC levels [39]. However, probiotic supplementation did not affect pregnancy outcomes, including gestational hypertensive disorders [39]. Accordingly, another double-blind RCT did not find a considerable effect on the prevalence of pre-eclampsia in women with GDM [40]. In this study, 149 enrolled women were randomly assigned to receive a probiotic (*Lactobacillus salivarius*) or placebo capsule each day from diagnosis up to childbirth for 8 weeks [40].

Furthermore, a meta-analysis of nine RCTs with 695 pregnant women with GDM concluded that the prevalence of maternal gestational hypertensive disorders was not different between probiotic and placebo groups [41]. A subsequent meta-analysis of five RCTs including 402 pregnant women with GDM was performed to assess the effectiveness of probiotic or synbiotic supplementation against gestational hypertension for a period between the sixth and the eighth week of gestation in comparison with a placebo [42]. Probiotic or synbiotic administration affected neither systolic and diastolic blood pressure nor the probability of pre-eclampsia. However, the currently available surveys remain restricted, also presenting high heterogeneity between studies. Hence, future high-quality RCTs are supported to be performed [42]. In another recent meta-analysis including five RCTs with 1048 pregnant women affected by overweight or obesity, the results suggest that probiotics may enhance the probability of pre-eclampsia, including superimposed [43].

Based on the above evidence, it appears that probiotic or synbiotic supplementation may exert a beneficial impact on diseases like elevated blood pressure and pre-eclampsia if received as a preventive agent and for a prolonged period. This conclusion may be ascribed to the probable regular impacts of probiotics on the gut microbiome and improvement in the occurring situations. Moreover, surveys which administrated probiotics for a period longer than 8 weeks led to a higher decrease in systolic and diastolic blood pressure compared with surveys which utilized the same probiotic supplementation for less than 8 weeks. In addition, surveys which utilized various probiotic species in these supplements seemed to exert a more favorable effect on systolic and diastolic blood pressure. However, the recent meta-analysis studies did not confirm the favorable effect of probiotics supplementation against gestational hypertension and pre-eclampsia, highlighting the strong demand for performing more RCTs to establish more accurate and conclusive results.

### 3.2. Lipid Metabolism Dysregulation in Pregnancy

Lipid metabolism during pregnancy has crucial implications for both the fetus and the mother, while essential fatty acids and cholesterol are essential nutrients for normal fetus growth. During gestation, numerous physiological alterations happen which result in changes in lipid profiles of healthy, pregnant women. These alterations in lipid levels throughout gestation permit the appropriate nutrient support of the fetus, which indicates enhanced insulin tolerance in the mother [44]. Nevertheless, it has been supported that dyslipidemia in gestation is related with harmful gestational outcomes, influencing both the mother’s and newborn’s health. A recent observational survey including a population of 16,489 singleton pregnant women showed that in the third trimester of gestation, serum concentrations of total cholesterol (TC), triglycerides (TGs), low-density lipoprotein (LDL) cholesterol, and high-density lipoprotein (HDL) cholesterol were all significantly increased [45]. A tendency concerning elevated probabilities of pregnancy complications and adverse perinatal outcomes was also noted in the women with enhanced concentrations of TC, TGs, and LDL cholesterol, while reduced levels of HDL cholesterol were recorded [45]. The possible positive effects of probiotic supplements in preventing or co-treating lipid metabolism dysregulation during pregnancy are presented in Table 2.

A double-blind RCT, including 60 participants with GDM, primigravida at the age of 18–40 years, assessed the potential beneficial effects of a daily capsule which included three viable freeze-dried strains *Lactobacillus acidophilus* (2 × 10^9^ CFUs/g), *Lactobacillus casei* (2 × 10^9^ CFUs/g), and *Bifidobacterium bifidum* (2 × 10^9^ CFUs/g) for 6 weeks [46]. More to the point, probiotics administration decreased both serum TG and very LDL (VLDL) cholesterol levels in comparison with the placebo. However, lipid profiles were not considerably affected by probiotics administration [46]. Another clinical trial conducted with 48 pregnant women diagnosed with GDM resulted in considerably lower TG and VLDL cholesterol concentrations and a lower total/HDL cholesterol fraction in the probiotics group in comparison with the placebo group [47]. Simultaneously, HDL cholesterol concentrations were considerably elevated in the probiotics group compared to the placebo one [47].

Furthermore, another clinical trial included 60 pregnant women enrolled into two groups: a group treated with multiple probiotic species (*Lactobacillus acidophilus*, *Lactobacillus casei*, and *Bifidobacterium bifidum*; 2 × 10^9^ CFUs/g each) and a group which consumed a placebo starting at the 9th week of pregnancy for a period of three months [48]. Significant decreases in serum TG levels in the supplemented women were noted compared to the placebo group [48]. The same research group then performed a double-blind RCT with 87 pregnant women with GDM who were enrolled into three groups to take either probiotics (8 × 10^9^ CFUs/day) or a placebo for 6 weeks [49]. Remarkably, vitamin D in addition to probiotic co-administration considerably decreased TG and VLDL cholesterol concentrations and the HDL/total cholesterol fraction [49]. Moreover, HDL cholesterol levels were substantially higher in the probiotic group than in the placebo group [49]. A previous clinical trial included 70 participants with GDM allocated into two groups: the synbiotic and the placebo group [50]. Women in the synbiotic group consumed a capsule per day which included three viable and freeze-dried strains: Lactobacillus acidophilus, Lactobacillus casei, and Bifidobacterium bifidum (2 × 10^9^ CFUs/g each) in combination with 800 mg inulin for 6 weeks. It was found that synbiotic intake significantly reduced serum TG and VLDL cholesterol concentrations compared with the placebo group [50].

In another RCT, 90 participants with GDM were enrolled into two groups to receive either a synbiotic capsule in combination with fructooligosaccharides (38.5 mg) each day, or a placebo for 6 weeks [37]. Considerable within-group elevation concerning HDL cholesterol and TC concentrations in the synbiotic group was noted. In addition, LDL cholesterol levels were significantly increased in the placebo group in comparison with the baseline of the survey [37]. Moreover, a double-blind RCT was also performed with 60 pregnant women with GDM who were divided into two groups to receive either a placebo or probiotic supplements (8 × 10^9^ CFUs/day) in combination with 200 μg/day selenium for 6 weeks [51]. Co-supplementation significantly decreased TG, TC, and LDL cholesterol concentrations compared to the placebo [51]. In a previous RCT applying a different approach, the enrolled participants were assigned into three survey groups: nutritional counseling with probiotics supplements or placebo in the first trimester of pregnancy and a control group [52]. Lipid profiles were not different amongst the studied groups throughout gestation [52]. However, TC and LDL cholesterol levels were lowered in both nutrition advice groups compared to the control group postpartum [52]. In this context, Asemi et al. previously performed a single-blind RCT among 70 pregnant women who were enrolled in the third trimester of gestation [53]. Participating women were allocated to receive 200 g/d of common yoghurt or a probiotic-enriched yogurt for 9 weeks [53]. The received probiotic-enriched yoghurt was a commercially accessible product prepared with the starter cultures of Streptococcus thermophilus and Lactobacillus bulgaricus, enhanced with probiotics cultures of two strains of Lactobacillus acidophilus and Bifidobacterium animalis with an overall dose of ≥1 × 10^7^ CFUs [53]. Even though the intake of probiotic yogurt for 9 weeks led to a considerable decrease in serum total, LDL, and HDL cholesterol concentrations and serum TG levels, no considerable changes were noted when comparing the probiotics with conventional yogurts concerning their impact on serum lipids levels. Within-group differences in the common yogurt group resulted in a significant decrease in HDL cholesterol concentrations and in a borderline decrease in serum TC concentrations [53].

Furthermore, another clinical trial included pregnant women with GDM receiving either a daily synbiotic capsule or a placebo for 6 weeks [54]. Synbiotic administration considerably reduced the logTG/HDL-C fraction with a moderate–low effect compared with placebo [54]. Another clinical trial assessed the potential effects of nutritional advice on blood lipid concentrations for the period of gestation as well as postpartum [55]. In this study conducted with 132 asymptomatic post-GDM women, the probiotic group consumed a cocktail of six probiotic strains from *Bifidobacterium* and *Lactobacillus* for a period of three months [55]. After 12 weeks of intervention, the probiotics group’s HbA1c, TC, and TG levels were significantly decreased, suggesting that multiple strains of probiotics could be advantageous for obtaining better lipid metabolic outcomes in post-GDM women by regulating intestinal dysbiosis [55].

In a recent meta-analysis including 11 studies, only TC levels were significantly reduced in pregnant women with GDM by adopting probiotics treatment [17]. In contrast, serum HDL and LDL cholesterol levels as well as TG concentrations were not affected by probiotics interventions [17]. Nevertheless, the sample sizes of the examined clinical trials were rather small, varying from 20 to 60 [17]. In another more recent meta-analysis including a total of 4865 pregnant women from 28 selected RCTs, it was shown that the consumption of probiotics notably reduced the mean VLDL levels, while no significant differences were found concerning TG, TC, and HDL cholesterol levels [20]. In a previous meta-analysis of eleven RCTs involving 719 participants with GDM, probiotic supplementation improved lipid profile biomarkers (TG and HDL-cholesterol levels) but had no impact on TC and LDL cholesterol levels [56]. Nevertheless, the clinical validity and generalizability of the previous findings suffered from several limitations which were ascribed to the high heterogeneity of the available clinical trials, the problematic characteristics of the primary and/or secondary outcomes, and/or the issue that most of the involving participants of this clinical study were from only one country, Iran [56]. Moreover, a meta-analysis including ten clinical trials with 1,139 pregnant women indicated that both TC and TG concentrations were considerably reduced by probiotics administration [57]. In another meta-analysis performed on 12 RCTs including 894 pregnant women with GDM, VLDL and total cholesterol levels showed a considerable decrease, whereas TG, HDL, and LDL levels were not affected [58]. Another meta-analysis including 10 surveys (n = 594 participants) showed no considerable differences between probiotics supplementation and placebo groups concerning the effects on TC, HDL and LDL cholesterol, and TG levels [59].

### 3.3. Gestational Weight Gain

Gestational weight gain (GWG) constitutes a crucial normal biological procedure which supports sufficient fetus development. Inadequate or excessive GWG has been related with an increased likelihood of harmful gestational outcomes [60]. Notably, pregnant women with higher GWG than the recommended are at higher risk of adverse pregnancy outcomes, including postpartum obesity, GDM, higher prevalence of caesarean delivery, or pre-eclampsia. Moreover, neonates experience harmful complications, like excessive body weight at childbirth and being large for their gestational age [61]. Additionally, participants presenting excessive GWG and their newborns exhibit permanent health troubles, such as obesity and an increased risk of diabetes mellitus type 2 and cardiovascular disorder [62]. It should be noted that the Institute of Medicine (IOM) has recommended an appropriate GWG to ensure the optimal health of the mothers and their newborns [63]. The IOM recommends little GWG for pregnant women, but only 28–32% of pregnant women exhibit adequate GWG, whilst approximately 25% are characterized by non-sufficient weight gain, and 50% of pregnant women gain more body weight than recommended by the IOM [64]. In this respect, women with overweight or obesity show the greatest incidence of excessive GWG that is tending progressively predominant amongst gestations globally [65]. The potential positive impacts of probiotics supplements in preventing or co-treating GWG during pregnancy are presented in Table 3.

A recent and well-designed double-blind RCT evaluated the effects of fish oil and/or probiotics supplementation on GWG and body composition [66]. In fact, 439 overweight women were divided into four groups: fish oil and placebo, probiotics and placebo, fish oil and probiotics, and placebo and placebo. Fish oil included 1.9 g docosahexaenoic acid (DHA) and 0.22 g and eicosapentaenoic (EPA) acid and probiotics included both *Lactobacillus rhamnosus* and *Bifidobacterium animalis* ssp. *lactis* [66]. Mean GWG as well as body fat mass/percentage were not affected by the above interventions [66]. Moreover, a clinical study enrolled pregnant women with diet-controlled GDM at 24–28 weeks of gestation who were assigned to take either probiotics supplements including *Bifidobacterium* and *Lactobacillus* or a placebo each day for one month [67]. After the one-month intervention, GWG between the enrolled pregnant women in the probiotic and the placebo group did not show any considerable difference [67]. In addition, another clinical survey included 60 pregnant women with GDM who did not receive any hypoglycemic treatment and were allocated to take a probiotics capsule including *Lactobacillus acidophilus*, *Lactobacillus casei,* and *Bifidobacterium bifidum* (2 × 10^9^ CFUs/g individually) or a placebo for one and a half month. Again, probiotics supplementation did not exert any substantial effect on GWG between intervention and placebo groups [38].

In addition, a clinical trial was conducted including 48 participants suffering from GDM who were divided into two groups to take either a probiotics capsule comprising *Lactobacillus acidophilus*, *casei*, and *fermentum* as well as *Bifidobacterium bifidum*, (2 × 10^9^ CFUs/g individually) or placebo for a period of one and a half months [47]. Probiotics supplementation did not also exert any substantial impact on GWG between the two groups [47]. A similar clinical trial including 60 participants with GDM who were enrolled into two groups to take probiotics supplementation or a matching placebo was also performed [46]. In this study, three viable freeze-dried strains, *Lactobacillus acidophilus* and *casei* as well as *Bifidobacterium bifidum* (2 × 10^9^ CFUs/g each), were administered in the probiotics group for 6 weeks. After analysis, GWG was not significantly different between the probiotics and placebo group [46].

Another RCT was conducted with 87 pregnant women with GDM who were divided into three groups receiving either vitamin D (50,000 IU/every for 2 weeks) plus probiotics (8 × 10^9^ CFUs/day), probiotics alone (8 × 10^9^ CFUs/day), or placebo for one and a half months. Probiotics supplements did not exert any substantial effect concerning excessive GWG [49]. In addition, a clinical trial conducted with 70 participants with GDM divided them into two groups, a synbiotic and a placebo group [50]. During this study, the assigned women received synbiotics (a daily capsule including *Lactobacillus acidophilus* and *casei* as well as *Bifidobacterium bifidum*, 2 × 10^9^ CFUs/g each, in combination with 800 mg inulin) for one and half months [50]. Accordingly, this survey did not observe any substantial difference between probiotics supplementation and placebo concerning GWG [50]. In a later RCT, 90 participants with GDM were divided into two groups to receive a synbiotic capsule each day consisting of *Lactobacillus acidophilus*, *Lactobacillus plantarum*, *Lactobacillus fermentum*, and *Lactobacillus gasseri* (1.5–7.0 × 10^9−10^ CFUs/g) plus fructooligosaccharides (38.5 mg), or placebo for one and a half months. Again, probiotics supplementation did not exert any substantial effect on GWG [37]. Moreover, a clinical survey including both overweight and obese participants administered probiotics (*Lactobacillus rhamnosus* and *Bifidobacterium animalis’* subspecies *lactis*) in the second trimester until the 28th week of pregnancy [68]. In contrast to the previous studies, this study found a significantly lower prevalence (32.5%) of excessive GWG in pregnant women receiving probiotics compared to the incidence (46%) of women receiving placebo [68]. In contrast, a recent meta-analysis including five RCTs, which all administrated diverse species of *Lactobacillus* and *Bifidobacterium*, did not find any significant difference between the effects of probiotics supplementation and placebo on excessive GWG in participants affected by overweight or obesity during gestation [43].

### 3.4. Preterm Birth

Preterm delivery constitutes one of the most common causes of perinatal mortality and morbidity globally. This pathological condition accounts for the majority of negative outcomes of gestation, constituting 70% of perinatal deaths and approximately 50% of all postnatal neurological problems, resulting in a substantial burden on society [69]. It has been speculated that probiotics may contribute to the prevention of preterm birth. In fact, probiotics may be protective against the procedures which could result in preterm labor by moving and killing pathogens by increasing the anti-inflammatory cytokines and by lowering the pH to create vaginal conditions suitable for beneficial bacteria [70]. The potential association of probiotic supplementation with the risk of preterm birth are reported in Table 4.

In support of the above evidence, in a prospective survey including 70,149 singleton pregnant women, Nordvist et al. showed that probiotic milk intake during early, but not prior to or during late gestation, was considerably related with a decreased likelihood of preterm birth [36]. Remarkably, both iatrogenic and spontaneous preterm birth (from the 22nd to 36th week of pregnancy) with spontaneous term controls (between the 39th and 40th week of gestation) were included in the preterm birth analysis leading to 34,458 cases [36]. This observational study provided substantial evidence for a relation between the timing of probiotic milk intake throughout gestation and the prevalence of preterm delivery [36]. In addition, probiotic use (*Lactobacillus acidophilus*, *Lactobacillus plantarum*, *Lactobacillus frementum*, and *Lactobacillus gasseri*) daily in 185 pregnant women with a gestational age higher than or equal to the 25th week did not elevate the frequency of preterm birth nor decrease the duration of gestation; however, the frequency of preterm birth was reduced in the oral probiotics group [71]. In this context, a retrospective study assessed the frequency of recurrent spontaneous preterm delivery in pregnant women who received probiotics containing *Clostridium butyricum*, *Enterococcus faecium*, and *Bacillus subtilis* [72]. More to the point, 51 pregnant women with a previous spontaneous preterm delivery who received probiotic supplements prior to the 14th week of pregnancy until delivery were enrolled [72]. The incidence of spontaneous preterm delivery in the next pregnancy among 255 pregnant women with a previous spontaneous preterm delivery that did not receive probiotics was compared with that in the probiotics group. Notably, the incidence of recurrent spontaneous preterm delivery was substantially decreased in the probiotics group (9.8%) compared to the non-probiotics group (31.0%), supporting evidence that probiotics may reduce the prevalence of recurrent spontaneous preterm birth [72].

Furthermore, a retrospective study assessed the impacts of probiotics supplementation on perinatal outcomes in pregnant women with an elevated risk of preterm birth [73]. Probiotics supplementation including *Streptococcus faecalis*, *Clostridium butyricum,* and *Bacillus mesentericus* was administered for the prevention of bacterial vaginosis or the treatment of constipation beginning at the 12.5th week of gestation until delivery. Oral probiotics supplements including *Clostridium* exerted a significant impact in preventing preterm birth prior to the 32nd week of gestation [73]. Moreover, a prospective survey examining data from a Norwegian cohort (n = 950 cases and 17,938 controls) reported a considerable preventive impact against spontaneous preterm birth (<37th gestational week) in women receiving elevated amounts of probiotic milk products, including *Lactobacillus acidophilus*, *Bifidobacterium lactis*, and *Lactobacillus rhamnosus* [74]. This study had several strengths, including its prospective methodology for gathering nutritional data and the food frequency questionnaire (FFQ) completed in the gestational period from the 17th to 22nd week prior to gestation until childbirth to minimize confounding impacts as a result of retrospectively answered questionnaires [74]. In addition, the sample size was large and represented women from all over Norway with various nutritional habits and a broad range of consumption incidences of probiotic products [74]. Another blind RCT was performed in Rwanda in pregnant women assigned to receive probiotic (*Lactobacillus rhamnosus* and *Lactobacillus reuteri*) or placebo capsules for four weeks [75]. Despite the small sample size and the absence of alterations in the microbiome, women in the placebo arm were considerably more likely to have a preterm childbirth [75]. Nevertheless, the small number of participants in this survey was certainly not adequate to find substantial changes in a reliable preterm labor incidence [75].

In contrast, a clinical study including participants affected by overweight or obesity assigned them to a probiotics group receiving *Lactobacillus rhamnosus* and *Bifidobacterium animalis* from the second trimester until delivery and to a placebo group [68]. This study did not observe any advantageous effect of probiotics administration on the risk of preterm birth [68]. Moreover, a clinical study evaluated whether the consumption of probiotic yoghurts containing *Lactobacillus acidophilus* and *Bifidobacterium lactis* may be associated with maternal and infant complications [76]. This study also showed that probiotics had no effect on the prevalence of preterm delivery in pregnant women affected by overweight and obesity with no diabetes throughout the pregnancy [76]. In another RCT, *Lactobacillus rhamnosus* and *Lactobacillus reuteri* (10^9^ CFUs each) or placebo were received for two months by women with <12 completed weeks of gestation until delivery [76]. Again, probiotics had no effect on the prevalence of preterm childbirth [77]. However, the recorded prevalence of preterm birth was very low in this study [77]. Accordingly, an RCT was performed in New Zealand. Participants presenting a personal or partner history of atopic disease were assigned between 14 and 16 weeks of pregnancy to take *Lactobacillus rhamnosus* (6 × 10^9^ CFUs) or placebo daily [78]. Once more, probiotics did not exert any impact on the prevalence of preterm birth [78]. A more recent RCT assessing potential differences between probiotics *lactobacilli* (*Lactobacillus rhamnosus* and *Lactobacillus reuteri*) and a placebo twice daily for 12 weeks was conducted with 86 asymptomatic pregnant women [79]. Again, probiotics did not exert any effect on the prevalence of preterm birth [79]. However, it should be noted that the recorded incidence of preterm birth was very low in the above survey [79].

A meta-analysis survey by Jarde et al. including five RCTs in which women with a singleton gestation consumed a probiotic supplement was performed [80]. This was the first meta-analysis which showed that receiving probiotics throughout gestation neither increased nor reduced the likelihood of preterm childbirth < 34 weeks (1017 women in 5 studies) or preterm childbirth < 37 weeks (2484 women in 11 studies) [80]. However, a severe disadvantage identified by this meta-analysis was the scheduling and period of the probiotics administration which was between the 1st and the 26th week. Even though in the majority of the included surveys the intervention was performed throughout the third trimester, some of the surveys administered the probiotics in the initial stages of gestation and certain studies merely focused on the last weeks of gestation. Thus, it is speculated that the duration of nutritional intervention may influence the results of this meta-analysis [80].

In a more recent meta-analysis, 21 studies were evaluated concerning the administration of probiotics supplements in pregnant women with an absence of a history of pathologies, focusing on the cases of preterm childbirth (<37th week of pregnancy), and it showed that that probiotic administration throughout gestation did not have any considerable effects on the risk of preterm delivery [81]. It should be noted that this absence of relation may be ascribed to the scheduling of the applied intervention, as many of the analyzed surveys administered probiotics supplements in the last 4–6 weeks of pregnancy up to childbirth or postpartum. In addition, it is notable that only 142 cases of preterm delivery were involved in this meta-analysis from a total of 2934 enrolled women, which indicates a low frequency of preterm birth in the included surveys (4.8%) in comparison with the worldwide estimations (10.6%) [81,82]. Taking into consideration the decreased recorded prevalence of preterm birth in the analyzed studies, upcoming surveys including a higher number of pregnant women and also containing more cases of preterm birth are highly recommended to assess the impacts of probiotics supplementation on preterm childbirth.

### 3.5. Vaginal Microbiota Disturbances

Dietary interventions, especially probiotics, have been considered to exert favorable impacts on the vaginal microbiome throughout gestation. More to the point, a recent RCT assessed the effects of fish oil (1.9 g DHA and 0.22 g EPA) and/or probiotic (*Lacticaseibacillus rhamnosus* and *Bifidobacterium animalis* ssp. lactis) dietary supplements on the vaginal microbiota of women affected by overweight or obesity throughout gestation [83]. A decreased quantity of possible pathobionts, such as *Ureaplasma urealyticum* in the fish oil group, *Ureaplasma*, Ureaplasma urealyticum, and *Prevotella disiens* in the probiotics group, and *Dialister invisus* and *Peptoniphilus timonensis* in the fish oil plus probiotics group, were detected [83]. Remarkably, the favorable effect of probiotics supplementation on vaginal microbiome components was enhanced by the decreased quantity of probable pathobionts, like *Prevotella*, *Peptoniphilus*, *Dialister,* and *Campylobacter,* from the initial months to the final months of gestation [83]. A previous pilot intervention survey involving 27 disease-free pregnant women documented that a mixture of *Lactobacillus*, *Bifidobacterium,* and *Streptococcus* strains given throughout the last three months of gestation was related with moderate regulation of the vaginal microbiome and cytokine release [84]. In fact, the probiotics intake led to the reduction in the pro-inflammatory chemokine Eotaxin, supporting a possible anti-inflammatory impact concerning vaginal immunity [84]. The potential beneficial effects of probiotic supplements in preventing or co-treating vaginal microbiome disturbances during pregnancy are presented in Table 5.

Furthermore, a pilot RCT determined whether oral intake of a mixture of two probiotics, *Lactobacillus acidophilus*, *Lactobacillus rhamnosus*, and bovine lactoferrin (Respecta^®^ complex), twice daily for 2 weeks may result in the detection of the consumed probiotics strains in the vagina [85]. Vaginal *Lactobacillus rhamnosus* and *Lactovacillus acidophilus* amounts were considerably elevated on days 14 and 21 [85]. In fact, on days 14 and 21, a considerable number of women showed elevated amounts of vaginal *Lactovacillus acidophilus*, while on days 7 and 21, a considerable number of women exhibited increased amounts of vaginal *Lactovacillus rhamnosus* [85]. More recently, a double-blind RCT with 40 women presenting signs or symptoms of vaginitis/vaginosis was performed [86]. Oral intake of a combination of lactobacilli including *Lactobacillus acidophilus* and *Lactobacillus rhamnosus*, in conjunction with bovine lactoferrin for a small period (i.e., 15 days), considerably increased the vaginal amounts of both *lactobacilli* species, *Lactobacillus acidophilus* and *Lactobacillus rhamnosus* [86].

In addition, a RCT was performed with 110 pregnant women at 35–37 weeks of pregnancy diagnosed by Group B *Streptococcus* (GBS) cultures who were allocated to orally consume two placebo capsules or two probiotic capsules (containing *Lactobacillus rhamnosus* and *Lactobacillus reuteri*) prior to bedtime until childbirth [88]. The above survey documented that oral probiotics *Lactobacillus rhamnosus* and *Lactobacillus reuteri* for 20 days may decrease vaginal and rectal GBS colonization in pregnant women [87]. This survey also supported that oral probiotics treatment should be received during the initial months of gestation to lower GBS colonization at 35–37 weeks of pregnancy and the above may decrease early-onset GBS infection, highlighting the demand for antibiosis treatment throughout labor. Additionally, it could help to overcome inadequate antibiosis treatment throughout labor in multiparous women, resulting in a decrease in admission rates to the newborn unit [87]. However, the above survey included some restrictions like a small sample size and a low degree of generalization across different regions and races [87]. Short-term intervention duration and a lack of data concerning the socio-economic status, nutritional behaviors, and the utilization of nutritional supplementation of the enrolled women were also limitations of this study [87]. Another RCT evaluated the capability of oral probiotics to eradicate vaginal GBS colonization throughout the third trimester of gestation [88]. Additionally, GBS-positive women were allocated to the verum group, treated with a dietary probiotics supplement of four viable strains of Lactobacillus twice daily for 14 days, or to a placebo group. The findings of this study did not reinforce the hypothesis that oral probiotics can eradicate GBS throughout gestation; however, a marginal correlation was found between increased GBS persistence and probiotics consumption [88].

Another clinical study was designed to compare probiotics intake per day (*Lactobacillus rhamnosus* and *Lactobacillus reuteri*) with placebo in a study sample of 66 asymptomatic pregnant women divided into a probiotic and a placebo group and presenting an Intermediate or Bacterial Vaginosis Nugent score at the 13th week of pregnancy [79]. This clinical trial found that probiotic Lactobacillus strains administration for a duration of 12 weeks during gestation did not lead to deleterious effects in women presenting low risk of preterm childbirth. The vaginal microbiome showed flux independently of probiotics consumption [79]. There is also a clinical study evaluating whether the vaginal colonization of lactobacilli in pregnant women could affect the risk of premature childbirth [89]. For this purpose, 40 pregnant women presenting increased risk of premature childbirth with normal vaginal microbiota (Nugent score ≤ 3) were enrolled to either consume two oral capsules daily of probiotics including 5 × 10^9^ CFUs *Lactobacillus rhamnosus* and *Lactobacillus reuteri* or no probiotics for a period of two months [79]. Treatments were subsequently crossed over for an extra 2 months [89]. Throughout the initial 2 months of treatment, *Lactobacillus rhamnosus* was found in one (5%) woman taking the probiotic treatment and two (11%) women receiving no treatment. In addition, *Lactobacillus rhamnosus* was found in the vagina samples of four (11%) women throughout probiotic treatment (of both groups), whereas *Lactobacillus reuteri* was not found in no samples. Overall, vaginal colonization of *lactobacilli* following per os treatment appeared to be low during pregnancy [89].

### 3.6. Depression and Anxiety

Depression and anxiety symptoms are quite usual amongst women during the perinatal phase. In fact, almost 18% of women living in high-income countries are diagnosed with depression symptoms of diverse severity during the prenatal phase and 19% during the postnatal phase [90]. Anxiety symptoms are also usual, experienced by approximately 19% of women during the prenatal phase and 14% during the postnatal phase [91]. Depression and anxiety symptoms appear to be varied concerning their persistence and timing, and they may affect in different ways both the mother’s transition to parenthood and the newborn’s growth [92]. The possible positive effects of probiotic supplements in preventing or co-treating depression and anxiety throughout gestation are presented in Table 6.

In a prospective, single-blind RCT, 42 women were enrolled to receive a probiotic (*Lactobacillus reuteri*) or placebo for 21 days [93]. Depression evaluated by the Edinburgh Postnatal Depression Scale (EPDS) was considerably improved in the enrolled women belonging to the probiotics group compared to those of the placebo group [93]. Accordingly, a double-blind RCT assessed the impact of *Lactobacillus rhamnosus* on mood postpartum in 380 pregnant women enrolled to consume a placebo or probiotic supplement each day from the 14th to 16th week of pregnancy until 6 months after delivery [94]. Mothers in the probiotic treatment group showed considerably decreased depression and anxiety scores assessed by modified versions of the EPDS and the State Trait Anxiety Inventory 6 (STAI6), respectively, compared to those in the placebo group [94]. However, a limitation of this survey concerns the fact that both the EPDS and STAI6 are screening rather than diagnostic tools. This study also used retrospective, self-reported mental health data, and thus recall biases may influence its results [94]. In addition, 80–90% of the enrolled pregnant women showed a history of asthma, eczema, or hay fever demanding medical remedies, and overall, the study population was non-deprived, non-obese, greatly educated, and predominantly European. Thus, the findings of the above survey cannot be generalized to other populations [94]. On the other hand, this survey had a strong methodology design as it was a double-blind RCT with a substantial group size [94].

Another RCT was performed with 264 overweight pregnant women who received probiotics (*Lactobacillus rhamnosus* and *Bifidobacterium animalis* ssp. *lactis*, 10^10^ CFUs each per capsule) and/or fish oil from early gestation until 6 months after delivery [95]. Depression and anxiety status was evaluated by the EPDS and anxiety subscale of the Symptoms Checklist (SCL-90) [95]. EPDS scores were elevated by 1.11 points in the fish oil plus probiotics group and reduced by 0.85 points in the fish oil plus placebo group. At one year postpartum, the fish oil plus placebo group showed decreased EPDS scores compared to the probiotics plus placebo group. On the other hand, no distinctions in SCL-90 scores in response to the above intervention were noted [95]. Thus, it was supported that probiotics and/or fish oil intervention may have a moderate effect on depression symptomatology but not on anxiety. However, dietary quality was inversely related with depression and anxiety symptomatology in the initial stages of gestation [95].

More recently, a controlled RCT in 40 pregnant women with previous depressive or anxiety symptomatology was performed [96]. Between the 26th and 30th week of pregnancy until childbirth, participating women received per os a probiotics multispecies combination or a placebo once daily [96]. After two months of the nutritional intervention, no considerable differentiation between the probiotics group and placebo group concerning depression symptomatology, anxiety, and stress was noted [96]. In this study, the EPDS and Leiden Index of Depression Sensitivity-Revised (LEIDS-R) for depression assessment were used. Pregnancy-Related Anxiety Questionnaire-Revised (PRAQ-R) and STAI for anxiety were also utilized. Thus, the differences in outcomes could be ascribed to the methodological differences between the above studies [96]. In this context, it should be noted that the study of Slykerman et al. [94] contained a considerably larger sample (n = 380) compared to the survey by Browne et al. [96] and used *Lactobacillus rhamnosus*. In contrast, the number of enrolled women in the study by Browne et al. were reasonably small (n = 40) and participants consumed a probiotics combination without the probiotic strain used by Slykerman et al. [94,96]. Moreover, the study population of Brown et al. contained a comparatively high proportion of women with greater socio-economic level, which decreases the generalizability of their findings [100]. Additionally, the survey by Browne also contained a comparatively high proportion of women with subclinical depression and anxiety symptomatology [96]. In this aspect, former systematic reviews and meta-analyses showed that lesser depression symptom intensity may directly be related with an enhanced placebo response, and therefore, it is probable that the placebo response was relatively elevated in the pilot study by Browne et al. [100].

Additionally, a clinical trial including a multinational population of 230 pregnant women affected by obesity was conducted by dividing participants either to receive probiotics (*Lactobacillus rhamnosus* and *Bifidobacterium lactis*) or placebo capsules up to the 36th week of gestation [97]. Probiotics supplementation had no considerable effect on depression, anxiety, or functional health and well-being scores from baseline to the 36th week of gestation, while no differentiation between the studied groups at 36 weeks was found [97]. In the above survey, depressive and anxiety symptoms were determined by the EPDS and STAI-6, respectively [97]. It must be taken into account that depressive and anxiety symptomatology was relatively low in the above survey, therefore establishing a floor impact, which could make it difficult to identify a favorable effect that could partially support its findings [97]. Thus, according to the above findings, the utilization of probiotics for mental health favorable effects could not be proposed at this time for pregnant obese women [97]. The conflicting evidence may be ascribed to the different strains of probiotics used. Remarkably, certain studies indicated that impacts of probiotic supplements may be associated with the different strains. Moreover, although most studies exploring probiotics and mental health outcomes have utilized strains from the Bifidobacterium or Lactobacillus genera of bacteria, there was considerable heterogeneity in the precise strains utilized [101].

A later meta-analysis of two RCTs including 512 pregnant women who were postnatally assessed by the EPDS did not show statistical difference between the probiotic and placebo groups. These findings indicated that maternal depression may be very complex, being influenced by diverse bidirectional factors [98]. Another meta-analysis included three RCTs with a reduced probability of biases involving 713 pregnant women [99]. Accordingly, the above survey showed no substantial differences between probiotics and control groups concerning depression scores in the final stage of follow-up [99]. Probiotics indicated a benefit, considering that the enrolled women’s scores were lower than the established cut-off for depressive symptoms; however, statistical significance was not obtained. In comparison with placebo, probiotic supplements in gestation decreased anxiety symptomatology. However, this benefit was not reflected by a decrease in the percentage of the enrolled women with scores higher than the determined cut-off for anxiety [99]. This meta-analysis was also restricted by the low number of RCTs assessing probiotics supplementation throughout gestation to improve mental health complications in the perinatal period and by the moderate quality of the currently available data for the outcomes assessed in the initial surveys [99].

### 3.7. Caesarean Section

Currently, the number of caesarean sections are continuously increasing globally, while several studies have already warned against their negative impacts for both the mothers’ and children’s health. Reasonably, caesarean sections, as usually happens in surgical processes, are related with several short- and long-term harmful gestational outcomes such as a higher probability of uterine rupture, ectopic gestation, fetus death, premature childbirth, and mental health disturbances [102,103]. Clinical studies evaluating the potential association of probiotic supplementation with the likelihood of caesarean section are depicted in Table 7.

Karamali et al. demonstrated that synbiotic capsules including *Lactobacillus acidophilus*, *Lactobacillus casei*, and *Bifidobacterium bifidum* (2 × 10^9^ CFUs/g each) together with 800 mg inulin for 6 weeks resulted in a significant decrease in cesarean section rates of the probiotic group compared to the placebo group in pregnant women with GDM but did not affect other pregnancy outcomes [104]. It should be noted that this study is currently under investigation and thus no conclusive results can be established. A previous study, which was another clinical trial, indicated that administering probiotics capsules containing *Lactobacillus acidophilus* and *casei* as well as *Bifidobacterium bifidum* (2 × 10^9^ CFUs/g each) for 6 weeks resulted in a substantial reduction in cesarean section rates of the probiotic group compared to the placebo group in participants with GDM who did not receive oral hypoglycemic agents [39]. On the contrary, another RCT included 439 pregnant women with a BMI ≥ 25 Kg/m^2^ and a mean gestational week of 13.9 ± 2.1 who were allocated into four interventional groups: fish oil plus placebo, probiotics (*Lactobacillus rhamnosus* and *Bifidobacterium animalis* ssp. *lactis*, 10^10^ CFUs each) plus placebo, fish oil plus probiotics, and placebo plus placebo [105]. In contrast to the previous studies, no considerable relation of probiotics supplementation with the kind of delivery was noted in the above survey [105].

Furthermore, in another RCT, a combination of probiotic bacteria derived by in vitro experimentations (*Bifidobacterium bifidum*, *Bifidobacterium lactis*, and *Lactococcus lactis*) was prenatally received by women with an enhanced probability of negative outcomes on their children (i.e., relatives’ history of allergic disorder) and newborns for the initial year of life. The above survey did not show any substantial relation of probiotic supplementation with the mode of delivery [106]. Accordingly, in a controlled RCT, overweight or obese women were allocated into a probiotic or conventional yoghurt group, receiving 100 g daily from the 24th week of pregnancy until childbirth [76]. The above survey did not support any considerable association of probiotic treatment with the mode of delivery [77]. Another RCT was also performed in New Zealand [78]. In this RCT, the participants with a previous history of atopic disorder were assigned between 14 and 16 weeks of pregnancy to receive *Lactobacillus rhamnosus* (6 × 10^9^ CFUs) or placebo daily until delivery. Again, the above survey showed no considerable association of probiotic treatment with the mode of delivery [78]. A more recent RCT has compared probiotics *lactobacilli* (*Lactobacillus rhamnosus* and *Lactobacillus reuteri*) with placebo in 86 asymptomatic pregnant women. More to the point, probiotics were received per os two times per day for 3 months and did not also show any effect on the mode of delivery [79].

More recently, a meta-analysis of five RCTs with an overall sample size of 402 participants with GDM documented no considerable difference between the probiotic and control group concerning the prevalence of caesarean section [42]. Another meta-analysis performed by Perez-Castillo et al. on 17 RCTs also demonstrated that probiotics treatment throughout gestation did not affect cesarean section rates [81]. Accordingly, a previous meta-analysis was performed on five controlled RCTs involving 1333 pregnant women who received probiotics supplements with Lactobacillus and/or Bifidobacterium during pregnancy [107]. Again, probiotics treatment did not exert any considerable impact on the incidence of caesarean section rates [107].

### 3.8. Other Adverse Pregnancy Outcomes

#### 3.8.1. Gastrointestinal Dysfunction

A recent clinical study evaluated whether probiotics may exert positive effects on hormonal alteration-related dysbiosis, which could affect the intestinal nervous system and gastrointestinal (GI) behavior throughout the initial stages of gestation [108]. This survey had a duration of 16 days including two phases of six daily administrated probiotics, mostly Lactobacillus, and 2 days with no probiotics. On a daily basis, several studies were performed to observe GI behavior and quality of life [108]. Probiotic supplements considerably lowered the intensity of vomiting, sickness, and constipation, and resulted in a better life quality [108]. In addition, probiotic intake significantly reduced the quantity of *Akkermansia muciniphila*, which are considerably associated with elevated vomiting [108]. However, certain findings of this study may be exploratory because of the small sample size (n = 32). Additional limitations of the above survey include the lack of sample randomization and not blinding pregnant women with a placebo [107]. Moreover, long-term probiotic impacts were not evaluated, possibly resulting in underestimated probiotic impacts [108].

Another clinical study evaluated probiotics supplementation combined with ursodeoxycholic acid in the therapy of intrahepatic cholestasis of gestation (ICG) [109]. In fact, 82 pregnant women with ICG were randomly assigned into an experimental group (a 380 mg probiotics intestinal-soluble capsule two times per day, together with a 90 mg ursodeoxycholic acid soft capsule three times daily) and a control group (a 90 mg ursodeoxycholic acid soft capsule three times daily), with 41 participants in each group [109]. The administration course was 4 months. Probiotic supplements together with ursodeoxycholic acid in the therapy of ICG efficiently improved liver function and the gut microbiome of the enrolled women, providing promising evidence for the improvement in the clinical diagnosis and therapy of this disorder [109].

#### 3.8.2. Immune System Dysregulation

In a multicenter allergy-prevention RCT, participants were enrolled at the 20th week of pregnancy and assigned to four survey clusters, one consuming both *Lactobacillus reuteri* oil drops and ω-3 PUFA capsules, the second receiving ω-3 PUFA supplements and placebo regarding *Lactobacillus reuteri*, the third receiving *Lactobacillus reuteri* and placebo concerning ω-3 PUFA, and the fourth group receiving placebo capsules and placebo oil drops [110]. After approximately 20 weeks of supplementation throughout the second half of pregnancy, the number of activated and resting regulatory T cells in peripheral blood were lower in the *Lactobacillus reuteri*-supplemented group [110].

#### 3.8.3. Mastitis

Acute mastitis constitutes one of the basic explanations why women interrupt breastfeeding, and medical treatment must simultaneously be utilized with attention. A meta-analysis containing six RCTs indicated that per os probiotic supplements throughout gestation could decrease the risk of mastitis [111]. After per os treatment with probiotic supplements, the amounts of bacteria in the milk of healthy women and mastitis patients were considerably decreased [111]. In two of the surveys containing 727 women, the intervention was introduced in the prenatal phase [112,113]. More to the point, Fernadez et al. documented that the administration of *Lactobacillus salivarius* prevented the infectious mastitis when per os received throughout late gestation in women with a history of infectious mastitis during previous gestations [112]. Specifically, this clinical survey was conducted on 108 women divided into a probiotic and a control group to assess the incidence of mastitis during the initial 3 months postpartum [112]. In a multicountry, multicenter, clinical study, 328 women were enrolled into a probiotic (*Ligilactobacillus salivarius*) or a placebo group [113]. The nutritional intervention began from the 35th week of gestation until the 12th week postpartum [113]. Women in the probiotic group had a 58% lower prevalence of experiencing mastitis [113]. In addition, Karlsson et al. performed a cohort survey of more than 50,000 women in Norway to determine if pregnant women receiving probiotic milk throughout gestation showed a decreased prevalence of breastfeeding problems, including mastitis [114]. The findings indicated that the consumption of probiotic milk (La-5, Bb12, and LGG) was directly associated with the prevalence of mastitis [114].

## 4. Discussion

In the last few years, there has been a continuously increasing number of clinical human surveys assessing the possible impacts of probiotics as a supplementary treatment approach against several adverse pregnancy outcomes. Most of them seem to provide promising results either as preventive factors or as co-treatment agents against pregnancy-related complications. Several studies used only one probiotic, while other studies examined a mixture of probiotics or a mixture of probiotics with prebiotics. In addition, there is diversity concerning the characteristics of the study populations as a considerable number of studies included pregnant women with GDM, a smaller number of studies focused on overweight and obese pregnant women, while certain studies examined healthy pregnant women. There is also diversity regarding the kind of received probiotic microorganisms, the dosage, and the period of the nutritional intervention, as well as the time of the beginning of the intervention during gestation. Thus, it is reasonable that all of the above variations may affect the exact effects of probiotics supplementation, rendering most of the currently available evidence inconclusive.

Furthermore, there are several contradictory findings even in study populations with the same characteristics concerning GDM, overweight/obese, or healthy participants. These contradictory results may be ascribed to the diverse eligibility criteria for choosing participants and the heterogeneity of the quality and the methodology of the existing clinical surveys, like differences in nationalities, baseline features and previous medical history of the enrolled pregnant women, diverse probiotics doses and bacterial species, and different kinds of childbirth, time periods, and incidence of probiotics supplementation. Despite the above limitations, there is promising evidence mainly for the potential beneficial effects of probiotics against lipid metabolism dysregulation and hypertensive disorders during gestation. Moreover, there are sufficient data to support that probiotics may positively affect vaginal microbiota. The currently available clinical studies examining the impact of probiotics on mental health disturbances and the prevalence of preterm birth remain quite controversial. On the other hand, the existing studies for the regulation of GWG and the risk of caesarean section have currently shown that probiotics cannot probably exert any positive effects. In any case, a mixture of probiotics and a higher administration duration seem to be more effective against adverse pregnancy outcomes. However, each study usually used different species of probiotics, rendering the existing evidence inconclusive.

In general, a probiotic dose of more than 10^6^–10^8^ CFUs/gr or 10^8^–10^10^ CFUs/gr seems to be sufficient and effective for most of the existing clinical studies. However, it must be emphasized that based on the World Gastroenterology Organization guidelines concerning probiotic supplements, there is currently no precise probiotics dosages which could be suggested [115]. This issue is mainly ascribed to the fact that even if some probiotics could exert a positive effect even at low dosages, some others could be needed at elevated dosages [115]. In addition, the effectiveness of a probiotic dosage could be not the same due to the probable different impact of each probiotic on specific disorders and health outcomes [115]. Moreover, the consensus on probiotics and prebiotics has implied that at least three months of intervention is needed to see considerable improvements concerning diverse adverse pregnancy outcomes and especially metabolism-related outcomes [116]. In this respect, it is questionable whether an interventional period of at least 3 months could be more effective in the case of probiotics and its impact on adverse pregnancy outcomes.

A considerable issue also deals with the fact that the doses of distinct microorganisms varied from 5 × 10^5^ CFUs to 5 × 10^10^ CFUs, whereas in some cases, the exact dose was unclear. Moreover, in some clinical studies, the doses were expressed as CFUs/g; however, the researchers did not state the mass of the probiotic product administered. There are also some studies which did not compare a probiotic/synbiotic administration with a placebo group, while certain studies utilized a group without treatment or routine care as a comparison. Concerning the route of probiotics administration, almost all clinical studies used an oral administration. Moreover, treatment vehicles included capsules in most of the currently available clinical studies. However, some studies used probiotic yoghurt or probiotic powder, and few studies used probiotic oil, tablets, or milk products. The enrolled pregnant women were usually treated with probiotic/synbiotic supplements throughout the third trimester of pregnancy and most of the existing clinical surveys administered mixtures of *Lactobacillus* and *Bifidobacterium* species, whereas other studies utilized only *Lactobacillus* species.

There is currently sufficient evidence suggesting that the effectiveness of probiotics is both strain-specific and disorder-specific. In this aspect, clinical guidelines and meta-analyses should acknowledge the significance of reporting outcomes in terms of both specific strain(s) of probiotics and the kind of disorder. There is substantial evidence implying that probiotic impacts are strain-dependent, and strain-specificity is frequently a poorly reported aspect of probiotics investigations [117]. Additionally, clinical surveys clearly designed to evaluate the safety of probiotic/synbiotic interventions are still lacking, highlighting the strong demand to conduct further research concerning the safety issues of probiotics/synbiotics [118]. Lastly, there are currently a small number of clinical surveys evaluating the potential advantageous effects of probiotics on gastrointestinal dysfunctions, immune system dysregulation, and mastitis, highlighting the need for further research on these diseases.

## 5. Conclusions

In the last decade, there has been a growing number of clinical surveys highlighting the beneficial impacts of probiotics supplements against several adverse pregnancy outcomes. However, the currently available clinical studies show several discrepancies and many of them include an insufficient number of pregnant women under study. Some of them have also certain limitations, while there is not adequate homogeneity concerning the study design methodologies and protocols. Thus, the currently existing evidence remains inconclusive, emphasizing the strong need to conduct further well-designed and well-organized clinical studies, including a more adequate sample size. Moreover, the clinical studies evaluating prebiotics are quite limited, highlighting the need to conduct more research on this issue. Lastly, there is a strong need to additionally carry out, in the future, prospective studies to examine whether there is a causal relation between probiotics supplementation and their benefits against adverse pregnancy outcomes.

## Figures and Tables

**Table 2:** Clinical surveys evaluating the impact of probiotics against lipid metabolism dysregulation in pregnancy.

Type of Study	Study Population	Probiotics Treatment	Main Findings	Ref.
Double-blind, placebo-controlled RCT	Pregnant women with GDM, n = 90	Synbiotic capsule including *Lactobacillus acidophilus*, *Lactobacillus plantarum*, *Lactobacillus fermentum*, and *Lactobacillus gasseri* (1.5–7.0 × 10^9−10^ CFUs/g) in combination with fructooligosaccharides (38.5 mg) for a period of 6 weeks during pregnancy.	Significant within-group elevations in HDL cholesterol concentrations in the synbiotic group were found. LDL cholesterol levels were significantly reduced.	[37]
Double-blind, placebo-controlled RCT	Pregnant women with GDM, n = 60	A daily capsule containing *Lactobacillus acidophilus* (2 × 10^9^ CFUs/g), *Lactobacillus casei* (2 × 10^9^ CFUs/g,) and *Bifidobacterium bifidum* (2 × 10^9^ CFUs/g) for 6 weeks.	Serum TG and VLDL cholesterol concentrations were reduced.	[46]
Double-blind, placebo-controlled RCT	Pregnant women with GDM, n = 48	*Lactobacillus acidophilus* (2 × 10^9^ CFUs/g), *Lactobacillus casei* (2 × 10^9^ CFUs/g), and *Bifidobacterium bifidum* (2 × 10^9^ CFUs/g) was administered daily for a period of 6 weeks during gestation.	Serum TGs, VLDL cholesterol levels, and total/HDL cholesterol ratio were reduced, while HDL cholesterol levels were increased.	[47]
Double-blind, placebo-controlled RCT	Pregnant women, n = 60	*Lactobacillus acidophilus, Lactobacillus casei,* and *Bifidobacterium bifidum* (2 × 10^9^ CFUs/g each) administration, beginning at the 9th week of pregnancy for a period of 12 weeks.	Serum TG levels were decreased.	[48]
Double-blind, placebo-controlled RCT	Pregnant women with GDM, n = 87	Three groups received either vitamin D (50,000 IU/every 2 weeks) in combination with probiotics (8 × 10^9^ CFUs/day), or just probiotics (8 × 10^9^ CFUs/day) for 6 weeks.	Vitamin D and probiotic co-supplements resulted in a considerable reduction in TG and VLDL levels, and HDL/total cholesterol ratio. HDL cholesterol levels were considerably reduced.	[49]
Double-blind, placebo-controlled RCT	Pregnant women with GDM, n = 70	*Lactobacillus acidophilus, Lactobacillus casei,* and *Bifidobacterium bifidum* (2 × 10^9^ CFUs/g each) in addition to 800 mg inulin for 6 weeks.	Synbiotic intake significantly decreased serum TG and VLDL cholesterol levels.	[50]
Double-blind, placebo-controlled RCT	Pregnant women with GDM, n = 60	Probiotics containing *Lactobacillus acidophilus*, *Bifidobacterium bifidum*, *Bifidobacterium lactis,* and *Bifidobacterium longum* (2 × 10^9^ CFUs/day each) in addition to selenium (200 μg/day) for 6 weeks.	Co-supplementation significantly decreased TG, TC, and LDL cholesterol levels.	[51]
Double-blind, placebo-controlled RCT	Pregnant women with GDM, n = 256	Nutritional advice and support regarding intake of probiotics or placebo and a control group from the initial 3 months of gestation until childbirth.	Lipid levels were not affected during gestation. TC and LDL cholesterol concentrations were reduced postpartum in both the nutritional advice groups in comparison with the control group.	[52]
Single-blind, controlled RCT	Pregnant women, n = 70	Probiotic yoghurt, including *Streptococcus thermophilus* and *Lactobacillus bulgaricus*, enhanced with a probiotic culture of *Lactobacillus acidophilus* and *Bifidobacterium animalis* with a min total of 1 × 10^7^ CFUs for 9 weeks.	Although the intake of probiotic yogurt led to a considerable decrease in serum total, LDL, and HDL cholesterol concentrations and serum TG concentrations, no considerable changes were observed between the probiotic yogurt group and the conventional yogurt group concerning the lipid profile.	[53]
Double-blind, placebo-controlled RCT	Pregnant women with GDM, n = 90	A daily synbiotic capsule, including *Lactobacillus. acidophilus* (5 × 10^10^ CFUs/g), *Lactobacillus plantarum* (1.5 × 10^10^ CFUs/g), *Lactovacillus fermentum* (7 × 10^9^ CFUs/g), *Lactobacillus Gasseri* (2 × 10^10^ CFUs/g), and 38.5 mg of fructooligosaccharides for 6 weeks.	Synbiotic administration considerably reduced logTGs/HDL-C ratio in comparison with the placebo group.	[54]
Double-blind, placebo-controlled RCT	Asymptomatic post-GDM women, n = 132	Six probiotic strains from *Bifidobacterium* and *Lactobacillus* (*Lactobacillus acidophilus*, *Lactobacillus casei* subsp., *Lactobacillus lactis*, *Bifidobacterium bifidum*, *Bifidobacterium infantis*, and *Bifidobacterium longum* at a dosage of 10^7^ mg each) for 12 weeks.	HbA1c, TC, and TG levels of the probiotics group were considerably decreased compared to the placebo group.	[55]
A meta-analysis of 11 RCTs	Pregnant women with GDM, n = 779	Eight RCTs used probiotics containing *Lactobacillus acidophilus*, *Lactobacillus fermentum*, *Lactobacillus casei, Lactobacillus reuteri*, *Lactobacillus salivarius*, *Lactobacillus delbrueckii bulgaricus*, *Bifidobacterium bifidum*, and *Streptococcus thermophilus*. Three RCTs used synbiotics containing *Lactobacillus acidophilus*, *Lactobacillus casei*, *Lactobacillus fermentum*, *Lactobacillus gasseria*, *Lactobacillus plantarum*, *Bififidobacterium bififidum*, *Bififidobacterium longum*, and *Bififidobacterium infantis*. The intake of probiotics/synbiotics per day varied from 1 × 10^9^ CFUs/capsule to 112.5 × 10^9^ CFUs/capsule. The period of intervention was from 4 to 8 weeks.	Only TC levels were significantly reduced after receiving supplemented probiotic therapy. Serum HDL and LDL cholesterol and TG concentrations were not affected by probiotics administration.	[17]
A meta-analysis of 28 studies	Pregnant women, n = 4865	A total of 19 different species were used as probiotics. *Lactobacillus acidophilus* (59.25%) and *Bifidobacterium lactis* (37.03%) were the two most commonly consumed probiotic species. The probiotic dose was determined as 4.63 × 10^7^ CFUs per day. Probiotic dosage in total lasted from the 4th to 40th week of gestation and in a few cases, 9 months after delivery.	Probiotics notably reduced the mean VLDL levels, while no significant differences were found concerning TG, TC, and HDL cholesterol levels.	[20]
A meta-analysis of 11 RCTs	Pregnant women with GDM, n = 719	Probiotics were administrated in eight clinical studies and synbiotics in three. *Lactobacillus* was administrated in all included clinical studies. *Bifidobacterium* was administrated in all included clinical studies except one. The duration of the probiotics administrations was 4–8 weeks.	Probiotic supplementation improved lipid profile biomarkers (TG and HDL cholesterol levels) but had no effects on TC and LDL cholesterol levels.	[56]
A meta-analysis on 10 RCTs	Five trials included pregnant women with GDM, and another five trials included pregnant women without GDM, n = 1139	Probiotics were daily administrated at a dosage ranging from 10^7^ CFUs/g to 10^10^ CFUs/g. A single probiotic species (*Lactobacillus* spp.) was administrated in three clinical surveys. Certain clinical surveys administrated a mixture of strains (*Lactobacillus* spp., *Bifidobacterium* spp., and others). The probiotics administration began from the first trimester of gestation in four studies and from the third trimester in six clinical studies. The period of probiotics administration was between the 4th and 24th week of gestation.	Considerable reductions in the TC and TG levels were noted in the probiotics groups.	[57]
A meta-analysis of 12 RCTs	Pregnant women with GDM, n = 894	Nine RCTs used probiotics supplementation, including *Lactobacillus* and *Bifidobacterium,* 2 × 10^10^ CFUs/g. Three RCTs used a synbiotic capsule (e.g., *Lactobacillus acidophilus, Lactobacillus casei,* and *Bifidobacterium bifidum,* 2 × 10^9^ CFUs/g each).	VLDL and TC levels exhibited a significant reduction, whereas TG, HDL, and LDL levels were not affected.	[58]
A meta-analysis of 10 RCTs	Pregnant women with GDM, n = 594	All surveys, excluding one, utilized multispecies probiotics that contained *Lactobacillus* and *Bifidobacterium*. Two surveys used *Streptococcus thermophilus*. The probiotic dosages ranged from 10^6^ to 112.5 × 10^9^ CFUs/capsule. The durations of the interventions were 4–8 weeks (mean = 6.5 weeks).	There was no considerable difference between probiotics supplementation compared to placebo concerning the impacts on TC, HDL and LDL cholesterol, and TG concentrations.	[59]

RCTs: randomized clinical trials, GDM: gestational diabetes mellitus, CFUs: colony-forming units, BMI: body mass index, TC: total cholesterol, VLDL: very low-density lipoprotein, LDL: low-density lipoprotein, HDL: high-density lipoprotein, and HbA1: hemoglobin A1.

**Table 3:** Clinical studies evaluating the effect of probiotics against gestational weight gain (GWG).

Type of Study	Study Population	Probiotics Treatment	Main Findings	Ref.
Double-blind, placebo-controlled RCT	Pregnant women with GDM, n = 90	A synbiotic capsule per day—including *Lactobacillus acidophilus*, *Lactobacillus plantarum*, *Lactobacillus fermentum*, and *Lactobacillus gasseri* (1.5–7.0 × 10^9−10^ CFUs/g)—with fructooligosaccharides (38.5 mg), or placebo for one and a half months.	Probiotics supplementation did not exert any considerable effect on GWG.	[37]
Double-blind, placebo-controlled RCT	Pregnant women with GDM, n = 60	*Lactobacillus acidophilus*, *Lactobacillus casei,* and *Bifidobacterium bifidum* (2 × 10^9^ CFUs/g each) for one and a half months.	Probiotics supplementation did not exert any considerable effect on GWG.	[39]
Meta-analysis of five RCTs	Overweight and obese pregnant women, n = 1048	The interventional duration differed from one month of gestation to six months postpartum. Every survey administrated diverse species of *Lactobacillus* and *Bifidobacterium*.	Probiotics supplementation did not exert any considerable effect on GWG.	[43]
Double-blind, placebo-controlled RCT	Pregnant women with GDM, n = 60	*Lactobacillus acidophilus* (2 × 10^9^ CFUs/g), *Lactobacillus casei* (2 × 10^9^ CFUs/g), and *Bifidobacterium bifidum* (2 × 10^9^ CFUs/g) for one and a half months.	There were no significant differences between probiotics and placebo groups concerning excessive GWG.	[46]
Double-blind, placebo-controlled RCT	Pregnant women with GDM, n = 48	Probiotic capsule including *Lactobacillus acidophilus, casei*, and *fermentum* as well as *Bifidobacterium bifidum* (2 × 10^9^ CFUs/g individually) for one and a half months.	Probiotics supplementation did not exert any considerable effect on GWG.	[47]
Double-blind, placebo-controlled RCT	Pregnant women with GDM, n = 87	Three groups receiving either vitamin D (50,000 IU/every 2 weeks) in combination with probiotics (8 × 10^9^ CFUs, daily), probiotics alone (8 × 10^9^ CFUs, daily), or placebo for 6 weeks.	Probiotics supplementation did not exert any considerable effect on GWG.	[48]
Double-blind, placebo-controlled RCT	Pregnant women with GDM, n = 70	*Lactobacillus casei* and *Bifidobacterium bifidum* (2 × 10^9^ CFUs/g each) plus 800 mg inulin for one and a half months.	No significant difference between probiotics and placebo groups concerning GWG was noted.	[50]
Double-blind, placebo-controlled, RCT	Overweight pregnant women, n = 439	Four intervention groups: fish oil + placebo, probiotics + placebo, fish oil + probiotics, and placebo + placebo. Fish oil contained 1.9 g DHA and 0.22 g EPA. Probiotics contained both *Lacto-bacillus rhamnosus* and *Bifidobacte-rium animalis* ssp. (10^10^ CFUs per capsule). The intervention was carried out between the initial visit in early gestation and childbirth.	Probiotics did not influence mean GWG or body fat mass/proportion.	[59]
Double-blind, placebo-controlled RCT	Overweight pregnant women, n = 439	Two fish oil capsules (a total of 2.4 g of n-3 PUFA including 1.9 g DHA and 0.22 g EPA) and one probiotic capsule (*Lactobacillus rhamnosus* and *Bifidobacterium animalis* ssp. *lactis*, individually 10^10^ CFUs per capsule) every day from 13.9 ± 2.1 week of gestation until delivery.	The mean GWG or the body fat mass/percentage were not considerably affected by probiotics administration.	[66]
Double-blind, placebo-controlled RCT	Pregnant women with diet-controlled GDM, n = 57	Probiotic supplements containing *Bifidobacterium* and *Lactobacillus* (2 × 10^9^ CFUs/g each) received each day for one month.	Probiotics supplementation did not exert any considerable effect on GWG.	[67]
Double-blind, placebo-controlled RCT	Overweight and obese pregnant women, n = 411	*Lactobacillus rhamnosus* and *Bifidobacterium animalis’* subspecies *lactis* at a daily dosage of >1 × 10^9^ CFUs each from 16 weeks of gestation until delivery.	A prevalence of 32.5% of women in the probiotics group had excessive GWG, which was significantly lower than the relevant prevalence (46%) of the participants in the placebo group.	[68]

RCTs: randomized clinical trials, GDM: gestational diabetes mellitus, CFUs: colony-forming units, DHA: docosahex-388 aenoic acid, EPA: eicosapentaenoic acid, and GWG: gestational weight gain.

**Table 4:** Clinical studies evaluating the potential association of probiotic supplementation with the risk of preterm birth.

Type of Study	Study Population	Probiotics Treatment	Main Findings	Ref.
Observational prospective cohort study	Pregnant women nulliparous, n = 37,050	Probiotic milk products: product A containing *Lactobacillus acidophilus, Bifidobacterium lactis*, and *Lactobacillus rhamnosus* GG, and product B containing *Lactobacillus acidophilus* and *Bifidobacterium lactis* (10^8^ probiotic bacteria/mL).	Probiotic milk consumption during early, but not before or during late pregnancy, was considerably related with decreased risk of preterm birth.	[36]
Double-blind, placebo-controlled RCT	Pregnant women affected by overweight and obesity, n = 411	*Lactobacillus rhamnosus* and *Bifidobacterium animalis’* subspecies *lactis* administered at a daily dosage of >1 × 10^9^ CFUs from the second trimester until delivery.	Probiotics had no impact on the prevalence of preterm birth.	[68]
Double-blind, placebo-controlled RCT	Pregnant women, n = 185	Probiotics capsule (*Lactobacillus acidophilus, Lactobacillus plantarum, Lactobacillus frementum*, and *Lactobacillus gasseri*) or vaginal probiotic capsule (*Lactobacillus plantarum, Lactobacillus acidophilus, Lactobacillus rhamnosus,* and *Lactobacillus gasseri*) each day for 37 weeks during pregnancy.	Probiotic use did not significantly affect the frequency of preterm birth or the duration of gestation, but the frequency of preterm birth was lowered in the oral probiotic group.	[71]
Retrospective placebo-controlled cohort study.	Pregnant women with a previous spontaneous preterm delivery who received probiotics prior to 14 weeks of pregnancy, n = 50. Pregnant women with a previous spontaneous preterm delivery who did not take probiotics, n = 255.	Probiotics containing Clostridium butyricum (10 mg/tablet), *Enterococcus faecium* (2 mg/tablet), and Bacillus subtilis (10 mg/tablet) were administered prior to the 14th week of pregnancy. Two tablets were received three times daily (six tablets/day) until the 36th week of gestation.	The rate of recurrent spontaneous preterm delivery was considerably decreased in the probiotics group (9.8%) compared to the non-probiotics group (31.0%), supporting evidence that probiotics may reduce the rate of recurrent spontaneous preterm birth.	[72]
Retrospective placebo-controlled cohort study	Pregnant women with elevated risk of preterm birth, n = 121	Probiotics including *Streptococcus faecalis, Clostridium butyricum,* and *Bacillus mesentericus* were administered at a dosage of 3–6 g, starting at about 12.5 weeks until delivery.	Probiotics *containing Clostridium* exerted a significant effect on the prevention of preterm birth before the 32nd week of gestation.	[73]
Prospective placebo-controlled cohort study	Pregnant women, n = 23,822	Milk-based beverages containing probiotic *lactobacilli* (probiotic milk product A containing *Lactobacillus acidophilus*, *Bifidobacterium lactis*, and *Lactobacillus rhamnosus*; milk product B containing *Lactobacillus acidophilus* and *Bifidobacterium lactis)* at a dosage from 6.6 g/d (one per month) to 1600 g/d (≥8 times per day) from 4th–5th months of pregnancy until delivery.	A significant protective effect against spontaneous preterm delivery (<37th week of gestation) in women with a high intake of probiotic milk products.	[74]
Double-blind, placebo-controlled RCT	Pregnant women, n = 30	One billion each of *Lactobacillus rhamnosus* and *Lactobacillus reuteri* for one month.	Probiotics significantly decreased the prevalence of preterm birth in the probiotics group compared to placebo.	[75]
Double-blind, placebo-controlled RCT	Overweight and obese pregnant women, n = 128	Yoghurts containing *Streptococcus thermophilus* and *Lactobacillus delbrueckii* subsp. *Bulgaricus* at a dosage of 10^7^ CFUs/g and 5 × 10^8^ CFUs/g of *Lactobacillus acidophilus* and *Bifidobacterium lactis* starting from the 6th to 10th week of pregnancy until birth.	Probiotics had no impact on the prevalence of preterm birth.	[76]
Placebo-controlled, triple-blind, parallel group RCT	Pregnant women with <12 completed weeks of pregnancy, n = 320	Daily intake of one capsule containing *Lactobacillus rhamnosus* and *Lactobacillus reuteri* (1 × 10^9^ CFUs of each strain per capsule) for 8 weeks.	Probiotics had no impact on the prevalence of preterm birth.	[77]
Double-blind, placebo-controlled parallel RCT	Pregnant women with a history of atopic disorder, n = 423	*Lactobacillus rhamnosus* (6 × 10^9^ CFUs) daily from 14–16 weeks of pregnancy until birth.	Probiotics had no impact on preterm birth.	[78]
Double-blind, placebo-controlled parallel RCT	Pregnant women, n = 86	*Lactobacillus rhamnosus* (2.5 × 10^9^ CFUs) and *Lactobacillus reuteri* (2.5 × 10^9^ CFUs) twice per day for 3 months.	Probiotics had no impact on the incidence of preterm birth.	[79]
Meta-analysis of 16 RCTs	Pregnant women, n = 4001	Eight surveys utilized only one or more species of *Lactobacillus*, six studies used a combination of *Lactobacillus* and *Bifidobacterium* species, five surveys utilized used a combination of *Lactobacillus, Bifidobacterium*, and *Streptococcus* species, and one survey combined two *Bifidobacterium* species with *Lactococcus lactis*.	Probiotics during pregnancy neither increased nor decreased the risk of preterm birth < 34th week (1017 women in 5 surveys) or preterm birth < 37th week (2484 women in 11 studies).	[80]
Meta-analysis of 46 RCTs	Pregnant women, n = 8519	Combinations of *Bifidobacterium* and *Lactobacillus species, Lactobacillus* species only, or combinations of *Lactobacillus*, *Bifidobacterium*, and *Streptococcus* species. One survey used *Bifidobacterium* species only and two studies assessed other mixtures with different bacteria (*Propionibacterium* and *Lactococcus)*.	Probiotic supplementation during gestation did not exert any considerable impact on the risk of preterm birth.	[81]

RCTs: randomized clinical trials, GDM: gestational diabetes mellitus, CFUs: colony-forming units, DHA: docosahexaenoic acid, and EPA: eicosapentaenoic acid.

**Table 5:** Clinical surveys evaluating the effect of probiotics supplementation on vaginal microbiota disturbances in pregnancy.

Type of Study	Study Population	Probiotics Treatment	Main Findings	References
Double-blind, placebo-controlled RCT	Pregnant women without signs of vaginal infection whose vaginal samples had a Nugent score ≥ 4, n = 66	*Lactobacillus rhamnosus* GR-1 and *Lactobacillus reuteri* RC-14 (2.5 × 10^9^ CFUs of GR-1 and 2.5 × 10^9^ CFUs RC-14) for 12 weeks.	Probiotic’ administration of *Lactobacillus* strains GR-1 and RC-14 throughout gestation in women with decreased risk of preterm childbirth showed no adverse side effects from the 12th week of gestation until delivery. Probiotics intake was associated with flux irrespective of the vaginal microbiota.	[79]
Placebo-controlled RCT	Pregnant women affected by overweight or obesity, n = 228 (women in early pregnancy, n = 112, and in late pregnancy, n = 116)	Fish oil (1.9 g DHA and 0.22 g EPA) and/or probiotic (*Lacticaseibacillus rhamnosus and Bifidobacterium animalis ssp. Lactis*, 10^10^ CFUs each) dietary supplements throughout the pregnancy.	A decreased quantity of pathobionts, such as *Ureaplasma urealyticum* in the fish oil group, *Ureaplasma, Ureaplasma urealyticum,* and *Peptoniphilus disiens* in the probiotics group, and *Dialister invisus* and *Peptoniphilus timonensis* in the fish oil plus probiotics group, was found. A decreased quantity of possible pathobionts, like *Prevotella*, *Peptoniphilus, Dialister,* and *Campylobacter,* between the initial months and the last months of gestation was noted.	[83]
Pilot, placebo-controlled, non-RCT	Pregnant women without symptoms of vaginal or urinary tract infection, n = 27	A total of 900 billion viable lyophilized bacteria including four strains of *Lactobacillus* (*Lactobacillus paracasei*, *Lactobacillus plantarum*, *Lactobacillus acidophilus*, *and Lactobacillus delbrueckii* subsp. *bulgaricus*), three strains of *Bifidobacterium* (*Bifidobacterium longum*, *Bifidobacterium breve*, and *Bifidobacterium infantis*), and one strain of *Streptococcus thermophilus* for one month between the 33rd and 37th week of pregnancy.	Probiotics administration was related with the modulation of the vaginal microbiota and cytokine secretion. The probiotic intake resulted in the reduction in the pro-inflammatory chemokine Eotaxin, supporting a possible anti-inflammatory impact on vaginal immunity.	[84]
Pilot, placebo-controlled RCT	Pregnant women without vaginal infections within previous 12 months, n = 40	*Lactobacillus acidophilus* and *Lactobacillus rhamnosus* (5 × 10^9^ CFUs), and bovine lactoferrin twice daily for 2 weeks.	Several women exhibited elevated amounts of vaginal L. acidophilus on days 14 and 21. Several women showed enhanced amounts of vaginal *Lactobacillus rhamnosus* on days 7 and 21.	[85]
Double-blind, placebo-controlled RCT	Pregnant women with signs or symptoms of vaginitis/vaginosis, n = 40	*Lactobacillus acidophilus* and *Lactobacillus rhamnosus* (5 × 10^9^ CFUs), and bovine lactoferrin twice daily for 2 weeks.	Probiotics administration significantly increased the vaginal amounts of *lactobacilli* species, *Lactobacillus acidophilus* and *Lactobacillus rhamnosus*. The impact of such colonization was associated with the restoration of a normal Nugent score (values 0–3) and an attenuation in symptomatology of non-normal vaginal microbiota including itching and discharge.	[86]
Double-blind, placebo-controlled RCT	Pregnant women at 35-37 weeks of gestation diagnosed with Group B Streptococcus culture, n = 99	Two probiotic’ capsules including dried viable *Lactobacillus rhamnosus* GR-1 and *Lactobacillus reuteri* RC-14 (1 × 10^9^ viable cells of both strains) before bedtime were received during 35–37 weeks of pregnancy until delivery.	Probiotics administration reduced vaginal and rectal Group B *Streptococcus* colonization.	[87]
Double-blind, placebo-controlled RCT	Pregnant women presenting a positive Group B Streptococcus screening culture at 35–37 weeks of gestation, n = 99	Two probiotics capsules including dried viable *Lactobacillus rhamnosus* GR-1 and *Lactobacillus reuteri* RC-14 (1 × 10^9^ viable cells of both strains) before bedtime were received during 35–37 weeks of pregnancy until delivery.	There was no significant trend toward reduced Group B *Streptococcus* persistence after probiotic intake.	[88]
Open-label, crossover, placebo-controlled RCT	Pregnant women characterized by elevated risk of premature childbirth presenting typical vaginal microbiota (Nugent score ≤ 3), n = 38	Administration of two capsules per day including 5 × 10^9^ CFUs *Lactobacillus rhamnosus* and *Lactobacillus reuteri* or no treatment for two months during gestation. Probiotics administration was afterwards crossed over for a further two months.	*Lactobacillus rhamnosus* GR-1 was found in one (5%) woman during probiotics administration and two (11%) women without treatment. *Lactobacillus rhamnosus* GR-1 was found in the vaginal samples of four (11%) women during probiotics administration (of both groups) and *Lactobacillus reuteri* RC-14 was not detected in any samples. Vaginal colonization of lactobacilli after the per os treatment was decreased throughout gestation.	[89]

RCTs: randomized clinical trials, GDM: gestational diabetes mellitus, CFUs: colony-forming units, DHA: docosahexaenoic acid, EPA: eicosapentaenoic acid, and GBS: Group B Streptococcus.

**Table 6:** Clinical studies evaluating the possible favorable effects of probiotics supplements in preventing or co-treating depression and anxiety during pregnancy.

Type of Study	Study Population	Probiotics Treatment	Main Findings	Ref.
Single-blind, placebo-controlled RCT	Pregnant women, n = 42	*Lactobacillus reuteri* at a dose of 10^8^ × CFUs for 21 days.	Depression assessed by EPDS was considerably improved in the enrolled women of the probiotic group compared to those of the placebo group.	[93]
Double-blind, placebo-controlled RCT	Pregnant women, n = 380	*Lactobacillus rhamnosus* 6 × 10^9^ CFUs between the 14th and 16th week of gestation until 6 months after delivery.	Women in the probiotics group showed considerably decreased depression and anxiety scores assessed by modified versions of the EPDS and STAI6, respectively, compared to those in the placebo group.	[94]
Double-blind, placebo-controlled RCT	Pregnant overweight women, n = 264	Four intervention groups: probiotics plus placebo (i.e., placebo for fish oil), fish oil plus placebo (i.e., placebo for probiotics), fish oil plus probiotics, or placebo plus placebo (placebo for probiotics and placebo for fish oil). Probiotics: *Lactobacillus rhamnosus* and *Bifidobacterium animalis* ssp. *lactis*, 10^10^ CFUs each for every capsule. Fish oil capsules included 2.4 g of n-3 LC-PUFA, including 1.9 g DHA and 0.22 g EPA. Intervention period and dosage: one capsule of probiotics and two capsules of fish oil from initial weeks of gestation (mean: 13.9 ± 2.1 weeks of pregnancy) until 6 months postpartum.	EPDS scores were elevated by 1.11 points in the fish oil plus probiotics group and reduced by 0.85 points in the fish oil plus placebo group. At one year after delivery, the fish oil plus placebo group showed decreased EPDS scores compared to the probiotics plus placebo group. No changes in SCL-90 scores in response to the intervention were noted.	[95]
Double-blind, placebo-controlled RCT	Pregnant women with low-risk pregnancies and elevated depressive symptoms and/or anxiety, n = 40	Probiotics multispecies combination: *Bifidobacterium bifidum* W23, *Bifidobacterium lactis* W51, *Bifidobacterium lactis* W52, *Lactobacillus acidophilus* W37, *Lactobacillus brevis* W63, *Lactobacillus casei* W56, *Lactobacillus salivarius* W24, *Lactococcus lactis* W19, and *Lactococcus lactis* W58 (2.5 × 10^9^ CFUs each). Daily dosage of 2 g from the 26th to 30th week of gestation until delivery.	Probiotics groups had no considerable differences compared to the placebo group concerning depressive symptomatology, anxiety, and stress assessed by EPDS, LEIDS-R for depression, and PRAQ-R and STAI for anxiety.	[96]
Double-blind, placebo-controlled RCT	Pregnant obese women, n = 164	Probiotic capsules including *Lactobacillus rhamnosus* and *Bifidobacterium lactis* at a minimum dosage of 6.5 × 10^9^ CFUs per day from the 12th to 17th week of gestation until the 36th week of pregnancy.	No improvement concerning depression or anxiety, assessed by EPDS and STAI-6, respectively, or functional health and well-being scores was recorded for probiotics supplementation at the 36th week of gestation.	[97]
Meta-analysis of two RCTs	Pregnant women, n = 512; overweight women from the initial weeks of gestation until 6 months after delivery.	*Lactobacillus rhamnosus*, 6 × 10^9^ CFUs, between the 14th and 16th week of gestation until 6 months after delivery. Four intervention groups: probiotics plus placebo (i.e., placebo for fish oil), fish oil plus placebo (i.e., placebo for probiotics), fish oil plus probiotics, or placebo plus placebo (placebo for probiotics and placebo for fish oil) groups. Interventional duration: from initial weeks of gestation (mean: 13.9 ± 2.1 gestational weeks) until 6 months after delivery.	EPDS for mothers showed no statistical difference between probiotic and placebo groups.	[98]
Meta-analysis of three RCTs	A total of 713 pregnant women (involving a low risk of bias)	One probiotic capsule per day, including *Lactobacillus rhamnosus* at a dosage of 6 × 10^9^ CFUs, from assignment until 6 months postpartum. Intervention period and dosage: one capsule of probiotics and two capsules of fish oil from early pregnancy (mean: 13.9 ± 2.1 gestational weeks) until 6 months postpartum.	There were no substantial differences between the probiotics and control groups concerning depression scores at the end of follow-up.	[99]

RCTs: randomized clinical trials, GDM: gestational diabetes mellitus, CFUs: colony-forming units, DHA: docosahexaenoic acid, EPA: eicosapentaenoic acid, EPDS: Edinburgh Postnatal Depression Scale, STAI6: State Trait Anxiety Inventory 6, SCL-90: subscale of Symptoms Checklist, LEIDS-R: Leiden Index of Depression Sensitivity-Revised, and PRAQ-R: Pregnancy-Related Anxiety Questionnaire-Revised.

**Table 7:** Clinical studies evaluating the probable association of probiotic supplements with the probability of caesarean section.

Type of Study	Study Population	Probiotics Treatment	Main Findings	Ref.
Double-blind, placebo-controlled RCT	Pregnant women with GDM, n = 60	Synbiotic capsules containing *Lactobacillus acidophilus*, *Lactobacillus casei*, and *Bifidobacterium bifidum* (2 × 10^9^ CFUs/g each) together with 800 mg inulin for 6 weeks.	A substantially higher reduction in cesarean section rates in the synbiotic group compared to the placebo group was recorded.	[39]
Meta-analysis of 5 RCTs	Pregnant women with GDM, n = 402	Probiotic or synbiotic supplementation (*Lactobacillus, Bifidobacterium*, and *Streptococcus* species) at a dose range from 1 × 10^9^ to 5 × 10^10^ lasting from 6 to 8 weeks.	Probiotic/synbiotic supplementation did not affect the prevalence of caesarean section compared to the control group.	[42]
Double-blind, placebo-controlled RCT	Overweight and obese pregnant women, n = 128	Yoghurts contained *Streptococcus thermophilus* and *Lactobacillus delbrueckii subsp. Bulgaricus* at a dosage of 10^7^ CFUs/g and 5 × 10^8^ CFUs/g for *Lactobacillus acidophilus* and *Bifidobacterium lactis* starting between the 6th and 10th week of pregnancy until childbirth.	There was not any significant association between probiotic treatment and mode of delivery.	[76]
Double-blind, placebo-controlled parallel RCT	Pregnant women with a previous atopic disease, n = 423	*Lactobacillus rhamnosus* HN001 (6 × 10^9^ colony-forming units) daily from the 14th to 16th week of gestation until delivery.	There was not any significant association between probiotic treatment and mode of delivery.	[78]
Double-blind, placebo-controlled RCT	Pregnant women without symptomatology of vagina infections whose vaginal samples had a Nugent score ≥ 4, n = 66	*Lactobacillus rhamnosus* GR-1 and *Lactobacillus reuteri* RC-14 (2.5 × 10^9^ of GR-1 and 2.5 × 10^9^ RC-14) for 12 weeks.	Probiotics did not show any effect on the mode of delivery.	[79]
Meta-analysis of 17 RCTs	Pregnant women, n = 3445	Combinations of *Bifidobacterium* and *Lactobacillus species, Lactobacillus* species only, or mixtures of *Lactobacillus, Bifidobacterium*, and *Streptococcus* species. One study used *Bifidobacterium species* only and two studies evaluated other mixtures with various bacterial genera (*Propionibacterium* and *Lactococcus*).	Probiotics supplementation throughout gestation did not affect cesarean section rates.	[81]
Double-blind, placebo-controlled RCT	Pregnant women with GDM, n = 60	A probiotic capsule including *Lactobacillus acidophilus*, *Lactobacillus casei,* and *Bifidobacterium bifidum* (2 × 10^9^ CFUs/g each) for 6 weeks.	A substantially higher reduction in cesarean section rates in the probiotic group compared to the placebo group was recorded. However, this study is currently under investigation and thus no conclusive results can be established.	[104]
Double-blind, placebo-controlled RCT	Overweight or obese pregnant women, n = 439	Fish oil plus placebo, probiotics (*Lactobacillus rhamnosus* and *Bifidobacterium animalis* ssp. *lactis*, 10^10^ CFUs each) plus placebo, fish oil plus probiotics, and placebo plus placebo from 13.9 ± 2.1 week of pregnancy until childbirth.	There was not any significant association between probiotic treatment and mode of delivery.	[105]
Double-blind, placebo-controlled RCT	Pregnant women with a positive family history of allergic disease, n = 102	*Bifidobacterium bifidum*, *Bifidobacterium lactis*, and *Lactococcus lactis* (10^9^ CFUs each), were received throughout the final six weeks of gestation.	There was not any significant association between probiotic treatment and mode of delivery.	[106]
Meta-analysis of 5 RCTs	Pregnant women, n = 1333	*Lactobacillus* and/or *Bifidobacterium* (10^9^ CFUs) administration mainly from the 32nd to 36th week of gestation.	No significant effect on the prevalence of caesarean section was observed after probiotic administration.	[107]

RCTs: randomized clinical trials, GDM: gestational diabetes mellitus, CFUs: colony-forming units, DHA: docosahexaenoic acid, and EPA: eicosapentaenoic acid.

## Data Availability

The data of the present study are available upon request to the corresponding author due to the privacy policy.
